# Strained alkyne functionalization of carbon dots enables selective photodynamic killing of Gram-positive bacteria

**DOI:** 10.1039/d6nr01923d

**Published:** 2026-08-07

**Authors:** Cristina M. Santi, Arthur Eley, Yuiko Takebayashi, Calum S. Haydon, James Spencer, M. Antognozzi, M. Carmen Galan

**Affiliations:** a School of Chemistry, University of Bristol Bristol BS8 1TS UK m.c.galan@bristol.ac.uk; b School of Physics, University of Bristol Tyndall Avenue Bristol BS8 1TL UK massimo.antognozzi@bristol.ac.uk; c School of Biochemistry and Biomedical Sciences, University of Bristol Biomedical Sciences Building Bristol BS8 1TD UK jim.spencer@bristol.ac.uk

## Abstract

Metal-free photoactive nanomaterials offer a promising strategy to address antimicrobial resistance, yet strategies enabling tuneable and efficient activity remain limited. Here, we introduce a materials-led approach to antibacterial photodynamic therapy by engineering carbon dots (CDs) with a strained dibenzocyclooctyne (DBCO) surface motif. Direct installation of the strained alkyne yields photoresponsive DBCO-CDs that exhibit potent, selective light-activated antibacterial activity. Under low-energy LED irradiation (395–405 nm), DBCO-CDs achieve >99.9% inactivation of Gram-positive bacteria, including *Staphylococcus aureus*, while remaining inactive in the dark and against Gram-negative bacteria. Mechanistic studies reveal that the intact strained alkyne is essential for activity, with non-strained-featuring analogues showing no efficacy. Reactive oxygen species (ROS)-trapping experiments implicate H_2_O_2_-mediated oxidative stress, while sub-cellular fluctuation imaging demonstrates rapid collapse of intracellular dynamics, consistent with metabolic failure. Notably, DBCO-CDs disrupt established biofilms, reducing biomass by 57% upon irradiation. Collectively, our findings establish strained-alkyne surface engineering as a powerful and modular strategy for imparting photodynamic functionality to carbon nanomaterials, advancing next-generation antimicrobial and antibiofilm technologies.

## Abstract 2

Strained-alkyne functionalised CDs enable selective photodynamic antibacterial activity. Under low-energy LED light, they achieve >99.9% killing of Gram-positive bacteria and reduce biofilm biomass by 57% *via* ROS-mediated oxidative stress.

## Graphical abstract

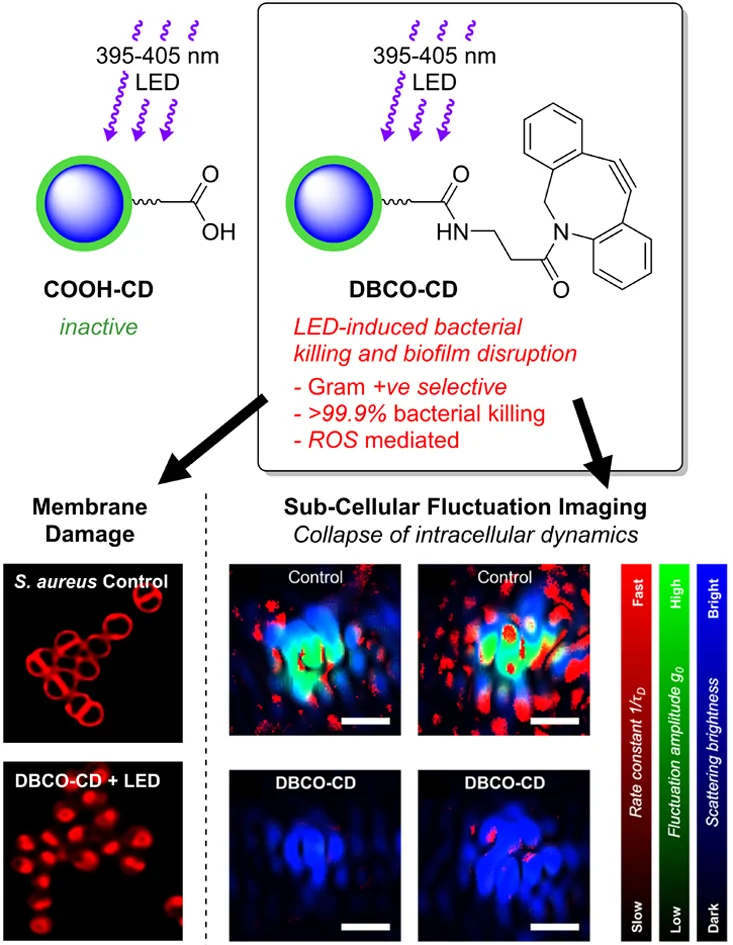

## Introduction

Bacterial antimicrobial resistance (AMR) is one of the leading public health threats of the 21^st^ century, with upwards of 1.9 million deaths attributed to AMR infections in 2019.^1,2^ Antimicrobial use exerts significant selective evolutionary pressure on microorganisms, driving the proliferation of advantageous mutations which afford the bacterium improved defence mechanisms against specific antimicrobial treatments. Unfortunately, this process occurs on short timescales. For example, the first *E. coli* strain able to resist penicillin was reported by Abraham and Chain in 1940, just a few years on from the discovery of penicillin by Fleming in 1928.^3^ Among Gram-positive bacteria, *Staphylococcus aureus* remains a globally dominant AMR threat, with recent burden analyses identifying it among the top bacterial causes of drug-resistant mortality; methicillin-resistant *S. aureus* (MRSA) alone accounts for over 100 000 deaths annually.^4^ Longitudinal genomic surveillance further reveals a significant and sustained rise in antibiotic-resistance gene prevalence in *S. aureus* over recent decades, highlighting its continued evolution under selective pressure.^4^

The ability of bacteria to develop AMR is even more relevant when cells are organised in biofilms, which are multicellular communities immersed in self-produced extracellular matrix, mainly made of polysaccharides, which allows adhesion to a surface. These structures protect bacteria from the external environment, increasing their tolerance to antibiotics and their resistance to immune system attacks, making them one of the key factors in chronic infections.^5^ It is therefore crucial to develop efficient alternative strategies for antibacterial therapy and biofilm treatment.

Antibacterial photodynamic therapy (APDT) has been recently rejuvenated as a potential broad-spectrum approach to fight drug resistant bacteria and biofilm-associated infections.^6^ APDTT relies on photo-induced, photosensitizer (PS)-promoted production of reactive oxygen species (ROS), able to inactivate bacteria and damage biofilms.^7–11^ As this method does not act on any one specific molecular target APDTAT is less likely to trigger drug resistance than the canonical antibacterial treatments.^12^ Metal- and carbon-based nanomaterials have been investigated as a new generation of photosensitisers due to their ability to produce ROS, driven by their unique surface properties such as surface plasmon resonance.^13^ Additionally, nanoparticles can be exploited as photosensitiser carriers to improve the inherent low stability and/or water solubility of PSs and to drive targeting to specific sites.^10,14,15^

Carbon dots (CDs) are a class of quasi-spherical carbon-based nanomaterials, under 10 nm in size, with unique photoluminescent properties, akin to semiconductor inorganic quantum dots (QDs).^16^ CDs benefit from excellent photo-stability, water solubility and inherently low cytotoxicity (unlike metal-based quantum dots), as well as expedient synthetic routes from readily available and affordable starting materials.^17–21^ Furthermore, their biocompatibility and large surface area favours the attachment of targeting molecules and biomolecules, which have resulted in their application in a wide range of fields including catalysis,^22^ sensing,^23^ antimicrobial,^24^ antifungal^25^ and antiviral^26^ agents, in gene delivery,^27–30^ cell imaging,^31,32^ photosynthesis augmentation,^33–35^ food preservation,^36–38^ cancer sensing,^39^ bioimaging,^40–42^ metal ion sensing,^43^ and as theranostics.^11,44,45^

Building on our interests in the development of robust methods for the synthesis of CDs, and their application as bioimaging and theragnostic agents, we recently disclosed the use of glucosamine-derived green-emitting CDs for bacteria labelling.^7^ Additionally, these amino-functionalised CDs could induce cell death upon LED irradiation (*λ* = 460 nm). However, despite their favourable photophysical properties and labelling efficiency, these CDs were unable to discriminate between Gram-positive and Gram-negative bacterial species.^7^

The photoluminescence, physicochemical properties, and ultimate biological function of carbon dots (CDs) are intrinsically linked to their molecular structure, which is governed by the choice of precursors, synthetic strategy, and surface functionalisation.^13,39,46,47^ Surface functional groups dictate charge distribution, hydrophilicity, and hydrogen-bonding capability, thereby strongly influencing interactions with biological membranes. Owing to the fundamental differences in cell envelope architecture between Gram-positive and Gram-negative bacteria, including the thick peptidoglycan layer of Gram-positive species and the lipopolysaccharide-containing outer membrane in Gram-negative species, tailored surface chemistries can be used to modulate electrostatic and more specific molecular interactions. These structural differences can be strategically exploited for the rational engineering of nanoparticles with specific surface functionalities that convey selective affinity toward specific bacterial classes.^48,49^

An attractive and biocompatible method for nanoparticle functionalization employs dibenzocyclooctyne (DBCO) groups, which enable copper-free strain-promoted cycloaddition reactions with azide-tagged biomolecules.^41,50–52^ Although strained alkynes such as DBCO are generally considered stable, previous research indicates that they may undergo degradation under oxidative, radical-rich, and acidic conditions, such as those encountered within endolysosomal compartments of immune cells.^53^ Despite the widespread use of the DBCO group in bioconjugation chemistry,^54^ the effects of such scaffolds on the photodynamic and biological labelling properties of carbon dots remain underexplored, especially in the context of antibacterial targeting and therapy. To address this gap, we embarked on a study investigating the bioactivity, specifically antibacterial activity, and photo-responsive properties of fluorescent CDs before and after DBCO conjugation. We hypothesised that installing DBCO directly onto the CD surface, given its high inherent reactivity, would not only provide a modular click-handle for modular functionalization, but might also modulate the photochemical properties of the nanomaterial, potentially enhancing ROS-mediated photodynamic killing.

## Results and discussion

**Scheme 1:**
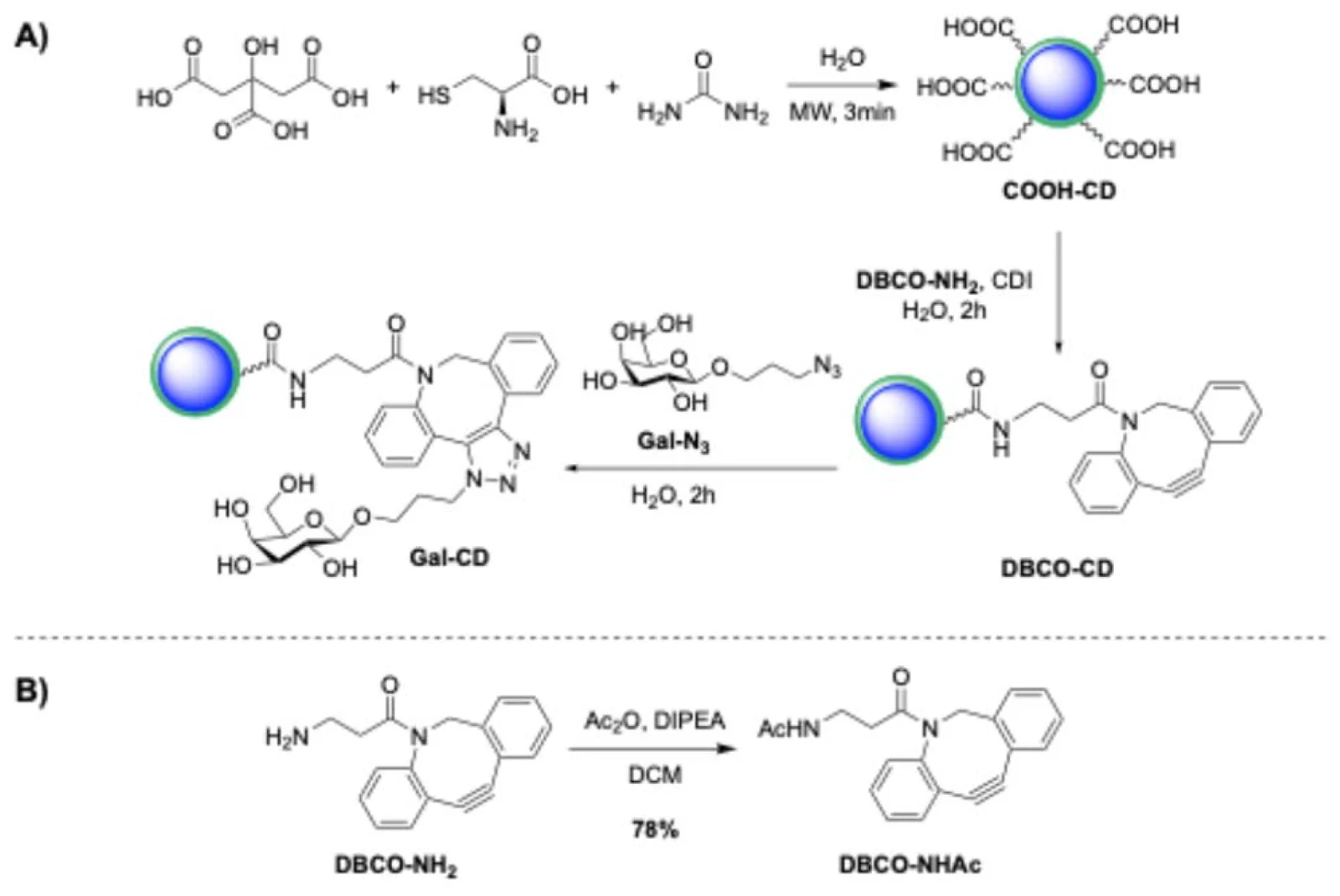
(A) Synthesis of COOH-CD, DBCO-CD, Gal-CD; (B) synthesis of DBCO-NHAc.^56^

To investigate the effect of DBCO surface-conjugation on CDs, and the potential of the resulting materials in antibacterial photodynamic therapy, we employed carboxylic acid-functionalised fluorescent carbon dots (COOH-CD), which were synthesised following a previously reported method from our lab.^42^ A rapid three-minute, microwave-assisted synthesis using citric acid, l-cysteine and urea afforded nanoparticles with an average diameter of 2.5 nm (Scheme 1). These CDs exhibit a double blue/green fluorescence profile, with excitation/emission at 340/450 nm and 391/499 nm, and quantum yields of 0.15 and 0.22, respectively (relative to quinine sulphate and coumarin 153 standards, respectively).^42^DBCO-CD were obtained *via* 1,1′-carbonyldiimidazole (CDI)-catalysed amide coupling between the native COOH-CD and dibenzocyclooctyne-amine (DBCO-NH_2_). The loading of DBCO on CDs was quantified *via*^1^H NMR spectroscopy by addition of a known amount of maleic acid as an internal standard (SI) and was determined to be 0.50 μmol DBCO per mg of DBCO-CD. In addition, biocompatible galactose-functionalised CDs (Gal-CD), synthesised *via* strain-promoted azide–alkyne cycloaddition of DBCO-CD and 1-*O*-(3-azidopropyl)-β-d-galactopyranoside^55^ and an acetamido analogue of the DBCO-moiety, DBCO-NHAc,^56^ were used as controls (Scheme 1). The quantum yield of functionalised CDs as in DBCO-CD was 70% lower than that of the parent COOH-CD as previously reported.^42^

**Fig. 1:**
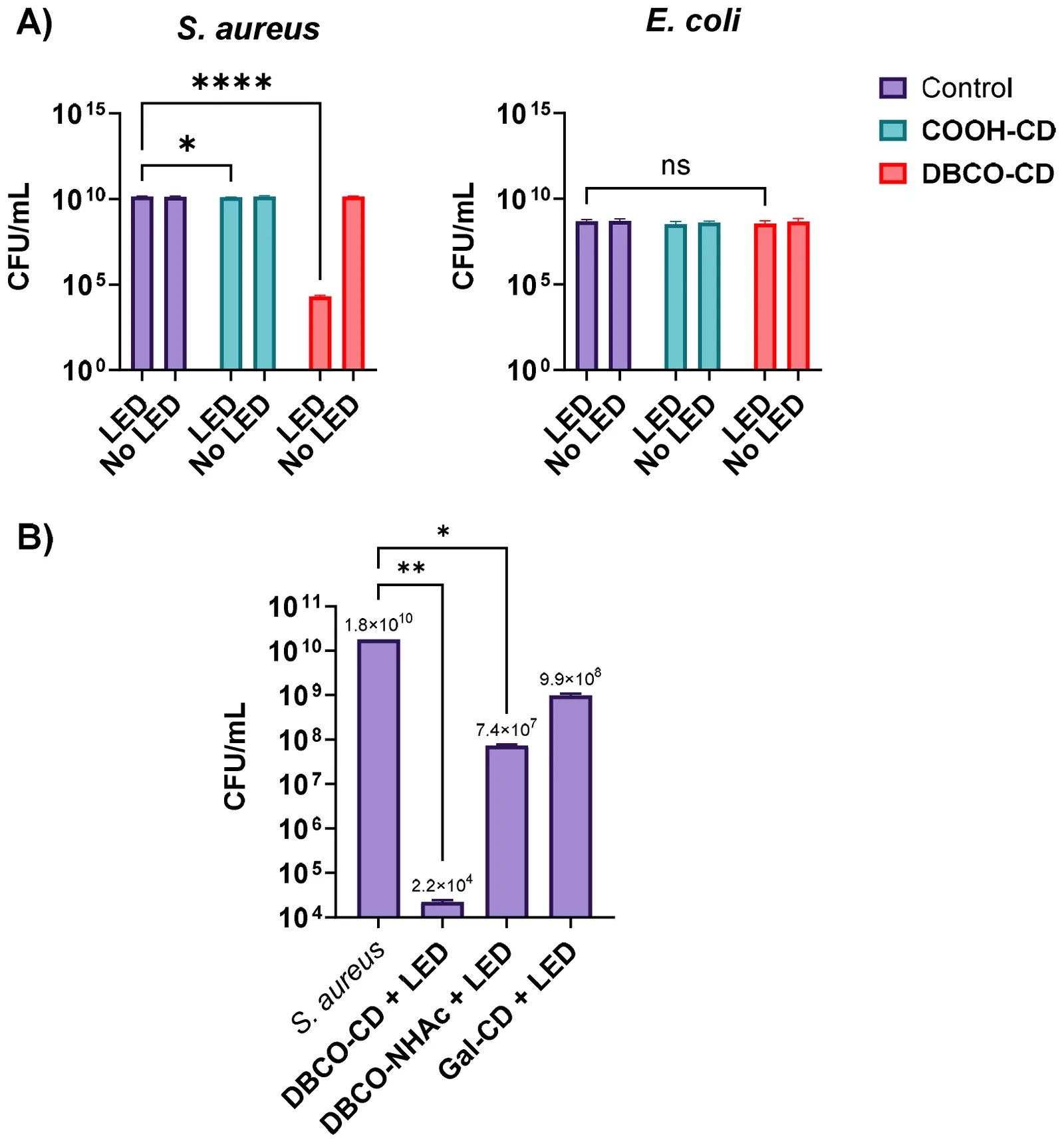
Photoactivated killing of *S. aureus* by DBCO-CDs. (A) Comparison of LED (*l* = 395–405 nm), COOH-CD ± LED and DBCO-CD ± LED treatment on the viability of *S. aureus* Newman (left) and *E. coli* BW25113 (right). (B) Effects of DBCO-CD + LED, DBCO-NHAc + LED and Gal-CD + LED treatment on *S. aureus* viability compared to an untreated and unirradiated control. Statistical significance assessed with the Mann–Whitney *U* test or two-way ANOVA. (*p* > 0.05 = ns, *p* < 0.05 = *, *p* < 0.01 = **, *p* < 0.001 = ***, *p* < 0.0001 = ****). Error bars indicate ± standard deviation of three replicates.

Preliminary experiments were aimed at evaluating the cytotoxicity of COOH-CD and DBCO-CD against two common pathogenic Gram-positive and Gram-negative bacterial species: *Staphylococcus aureus* Newman (*S. aureus*) and *Escherichia coli* BW25113 (*E. coli*). Bacteria (at starting concentrations of 10^9^–10^10^ CFU mL^−1^) were exposed to DBCO-CD and native unconjugated carboxyl-coated COOH-CD in PBS, both in darkness and with 395–405 nm LED irradiation for 90 minutes. This wavelength range was chosen to match the lower energy excitation wavelength of the particles. Any photoinduced antibacterial activity changes induced by the different CD-treatments were then evaluated by comparing numbers of viable bacterial cells remaining in samples treated with CDs, with and without LED irradiation to a control sample lacking both CD exposure and LED irradiation. The viability of *E. coli* and *S. aureus* cells exposed to LEDs in the absence of CDs did not decrease as a result of irradiation, allowing the attribution of any viability changes to effects caused by the introduction of the nanoparticles. Native carboxyl-functionalised COOH-CDs also had negligible effects on the viability of *S. aureus* and *E. coli*, both with and without LED irradiation. However, strikingly, after incubation of *S. aureus* with DBCO-CD, a 5.6-log reduction in viable cell numbers was observed after only 90 minutes of irradiation, corresponding to >99.9% killing, whereas viable cell counts in unirradiated samples showed no significant change. In contrast, the viability of *E. coli* irradiated in the presence of DBCO-CD remained unchanged (Fig. 1).

**Fig. 2:**
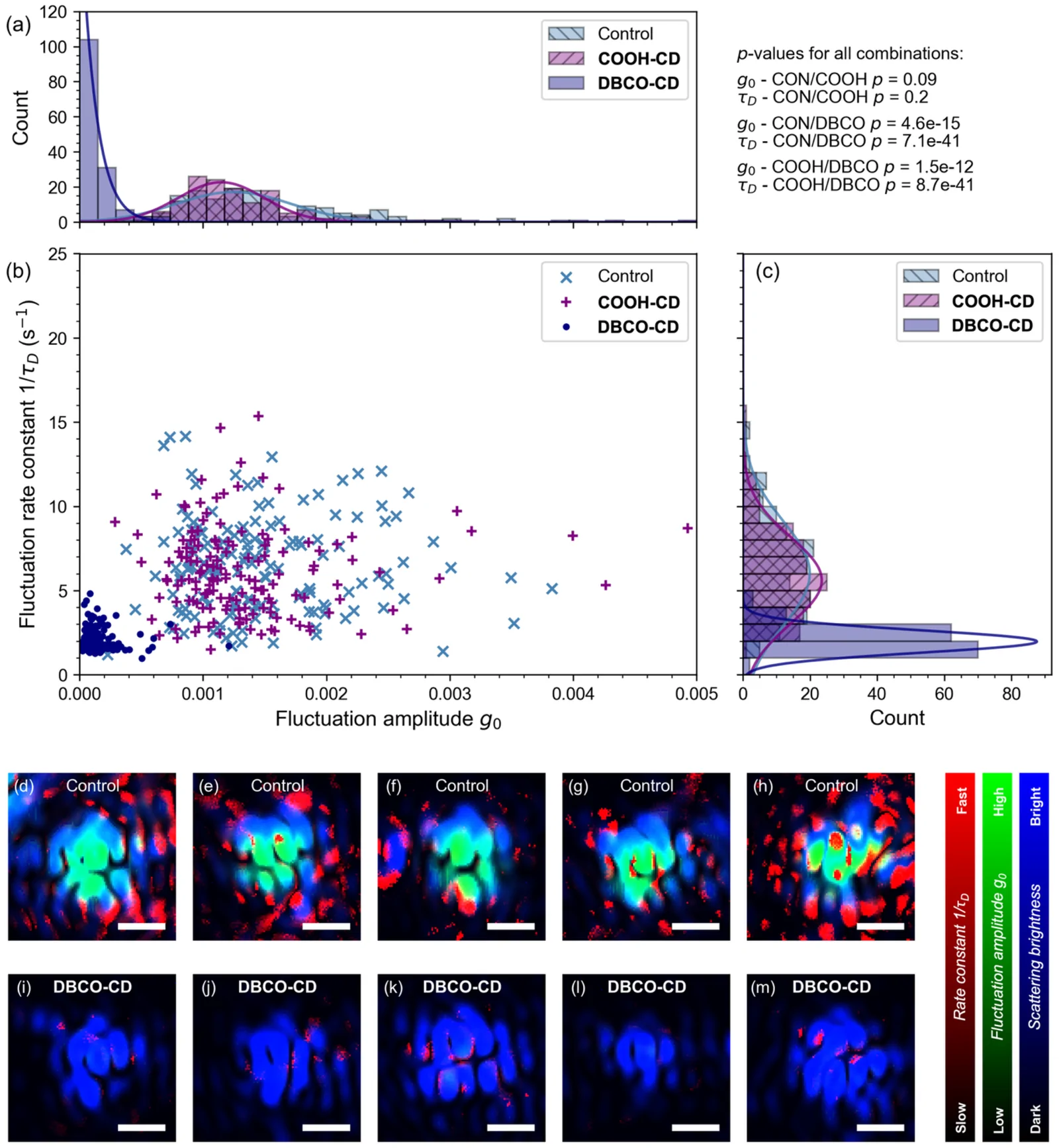
SCFI fluctuation data for *S. aureus* SH1000 exposed to COOH-CD, DBCO-CD, alongside the untreated control. All groups were exposed to 90 min of LED irradiation, including untreated control samples. (a) Comparison between distributions of *g*_0_ for the three suspensions. (b) Scatter plot of fluctuation amplitude (*g*_0_) *vs.* fluctuation rate (1/*τ*_D_) for control, COOH-CD and DBCO-CD treated suspensions. (c) Comparison between distributions of 1/*τ*_D_ for the three suspensions. Each sample group contains 150 cells, three triplicate repeats (each a group of 50 cells) that have been pooled together. *p*-Values when comparing each distribution given in the top right. False-colour RGB images shown for 5 control *S. aureus* cells (d–h) and 5 *S. aureus* cells exposed to DBCO-CD (i–m). Scale bar 0.75 µm.

Intrigued by these results, which imply DBCO functionalities on the carbon dot surface are key to their observed antibacterial activity, we decided to further investigate the photodynamic role of the strained alkyne moiety. To better understand the LED-promoted cytotoxicity mechanism of these DBCO derivatives at a single cell level, sub-cellular fluctuation imaging (SCFI), a technique recently developed by Antognozzi and co-workers was used.^57^ SCFI measures two parameters related to the activity within the cytoplasm of a single *S. aureus* cell. First, the rate constant, termed 1/*τ*_D_, is a measure of the characteristic timescale of sub-cellular dynamics. Second, the fluctuation amplitude, *g*_0_, quantifies the intensity variation relative to the mean scattering intensity.^57^ This parameter reflects the extent to which sub-cellular components are free to rearrange within the cytoplasm: the bacterial cytoplasm has been shown to exist in a glass-like state that is fluidised by metabolic activity,^58^ such that when metabolism is reduced or abolished, the cytoplasm approaches a glass transition, constraining the motion of intracellular components and causing both 1/*τ*_D_ and *g*_0_ to drastically decrease relative to metabolically active cells. For each bacterial cell, SCFI data are collected as a time stack of images recorded with a video camera. The analysis outputs a rate constant (1/*τ*_D_), a fluctuation amplitude (*g*_0_), and a mean scattered intensity for every pixel in the image. The median value of each metric, computed over the pixels within a region of interest inside the cell, is taken as representative of that cell. It is also possible to reconstruct false-colour images to examine the spatial variation of sub-cellular dynamics (Fig. 2d–m).

No statistically significant changes in rate constant (1/*τ*_D_) or fluctuation amplitude (*g*_0_) were seen between untreated controls and COOH-CD-treated cells, suggesting that COOH-CD exposure had no significant effect on *S. aureus* SCFI fluctuations (*p* = 0.2 and 0.09, respectively, Fig. 2). In contrast, DBCO-CD-treatment produced statistically significant reductions in both 1/*τ*_D_ and *g*_0_ (*p* = 7 × 10^−41^ and 5 × 10^−16^, respectively). When considering the changes to 1/*τ*_D_ and *g*_0_ in combination (Fig. 2b), exposure to DBCO-CD produced fluctuations at a level similar to the instrument's noise floor, and in this case, it is consistent with complete cell death.^57^ Comparison of the false-colour RGB images (Fig. 2d–m) shows that the reduction in 1/*τ*_D_ (red channel) and *g*_0_ (green channel) is uniform across DBCO-CD-treated cells, while the overall scattering brightness (blue channel) remains unchanged. For DBCO-NHAc and Gal-CD treatments, a detectable reduction in *S. aureus* SCFI fluctuations was observed (Fig. S2, in SI), although the effects were significantly smaller than those seen for DBCO-CD treatment (Fig. 2). Statistically significant reductions relative to the untreated controls were observed for *g*_0_ but not 1/*τ*_D_, suggesting that cells exposed to DBCO-NHAc and Gal-CD are less active when compared to the untreated controls. Exposure to DBCO-NHAc produced a greater reduction in *g*_0_ than in Gal-CD-treated cells, although neither treatment reduced fluctuations to the instrument's noise floor, suggesting the cells remain viable at the time of measurement.

To further probe the mechanism by which LED irradiation of DBCO-CD-treated *S. aureus* leads to cell death, we assess the possible involvement of reactive oxygen species. Standard colorimetric and fluorescence-based ROS assays could not be applied under our experimental conditions, since these methods rely on light-sensitive dyes, which would degrade upon 395–405 nm LED irradiation;^58,59^ in addition, the intrinsic fluorescence of the CDs would further interfere with the measurements. Consequently, an alternative strategy was adopted based on the report by Ramakrishnan *et al.*^60^ where sodium pyruvate (100 mM) functions as an H_2_O_2_ scavenger and protects *S. epidermidis* from ROS-mediated photoinactivation. We thus hypothesised that a reduction in DBCO-CD + LED cytotoxicity in the presence of sodium pyruvate would indicate inhibition of H_2_O_2_ formation and implicate ROS as a contributor to the antimicrobial activity observed.

**Fig. 3:**
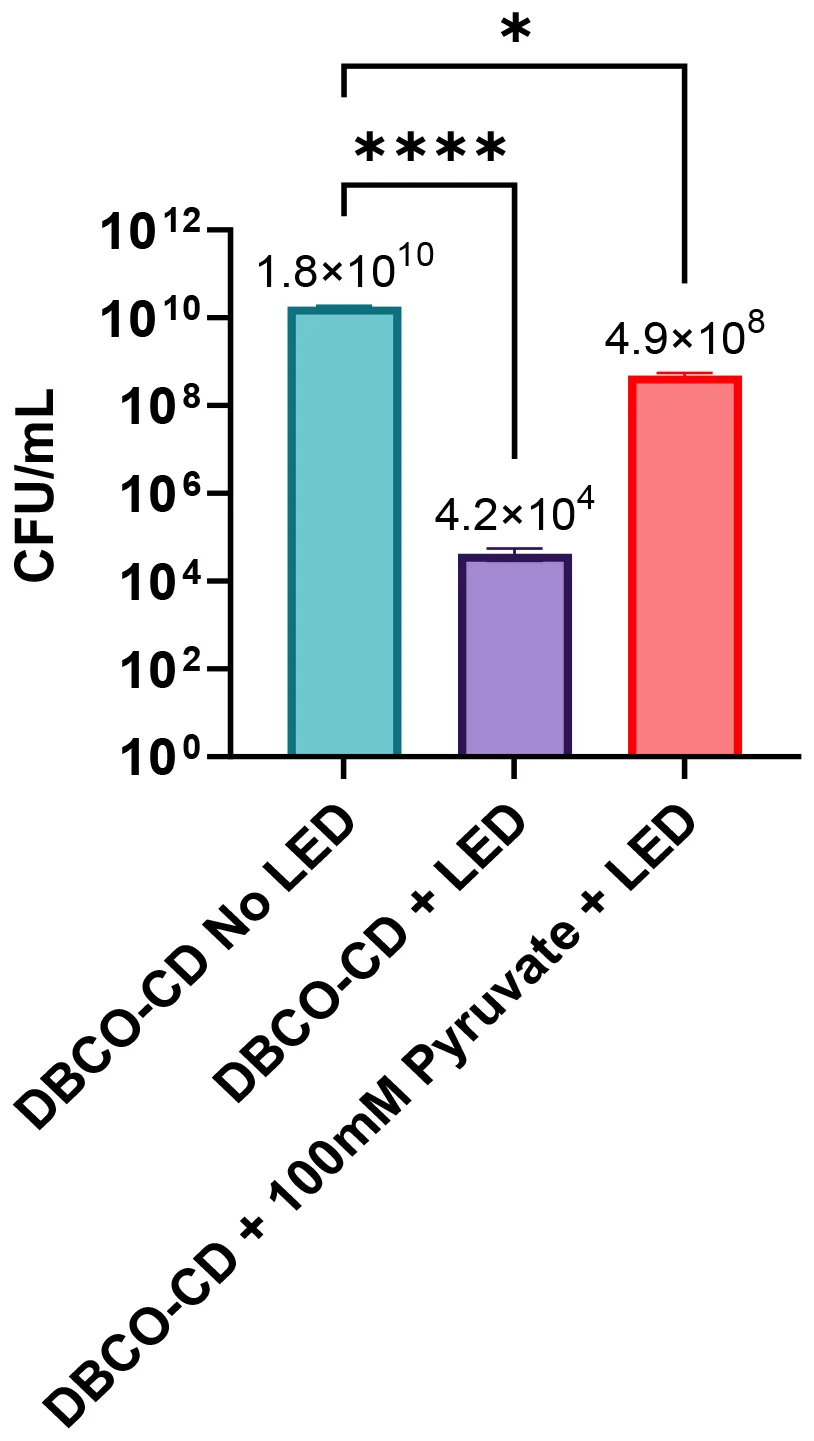
Effect of sodium pyruvate (H_2_O_2_ scavenger) on cytotoxicity of DBCO-CD/LED treatment after 90 min incubation with *S. aureus* Newman. Blue: DBCO-CD no LED; purple: DBCO-CD + LED; red: DBCO-CD + 100 mM sodium pyruvate + LED. Statistical significance assessed *via* one-way ANOVA. (*p* > 0.05 = ns, *p* < 0.05 = *, *p* < 0.0001 = ****). Error bars indicate ± standard deviation of replicates.

As shown in Fig. 3, in the absence of sodium pyruvate the treatment with DBCO-CD + LED led to a 5.6-log drop in CFU mL^−1^ compared to untreated controls (no CD, no LED). In the presence of sodium pyruvate, the reduction in CFU mL^−1^ decreased to 1.6-log, indicating that pyruvate mediated H_2_O_2_ scavenging attenuates the phototoxic effect. These results support ROS as an important contributor to the observed photodynamic antibacterial activity.

It is possible that irradiation-induced heating could account for the observed cytotoxicity of DBCO-CD. To test for this possibility, the temperature of solutions containing COOH-CD and DBCO-CD was monitored over time during LED exposure, and compared controls containing an equal volume of PBS only. An increase of <2 °C compared to the PBS control, after 90 minutes’ exposure, was registered for both the COOH-CD and DBCO-CD containing samples during irradiation (see SI/Fig. S3 for details). The temperature difference between the two treated samples was negligible. The small temperature increase of treated samples compared to untreated controls, together with the equivalent behaviours of bactericidal DBCO-CD and less cytotoxic native COOH-CD, suggests that thermal stress is unlikely to be the cause of DBCO-CD induced toxicity towards *S. aureus*.

We next sought to examine interactions of DBCO-CD with bacteria in more detail, beginning with an investigation of cellular uptake. Taking advantage of the inherent fluorescence of the CDs, *S. aureus* Newman cells were incubated with either COOH-CD and DBCO-CD for 1 h in darkness, then washed to remove any unbound nanomaterial, to determine whether DBCO-functionalisation of the CDs had an effect on cellular uptake. Fluorescence intensity was then quantified using a plate reader. An observable increase in measured fluorescence output was measured for cells treated with DBCO-CD compared to COOH-CD or untreated controls (see SI, Fig. S1). It is important to note due to the lower quantum yield of DBCO-CD*vs.*COOH-CD,^42^ it is likely that fluorescence measurements alone underestimate cellular uptake.

**Fig. 4:**
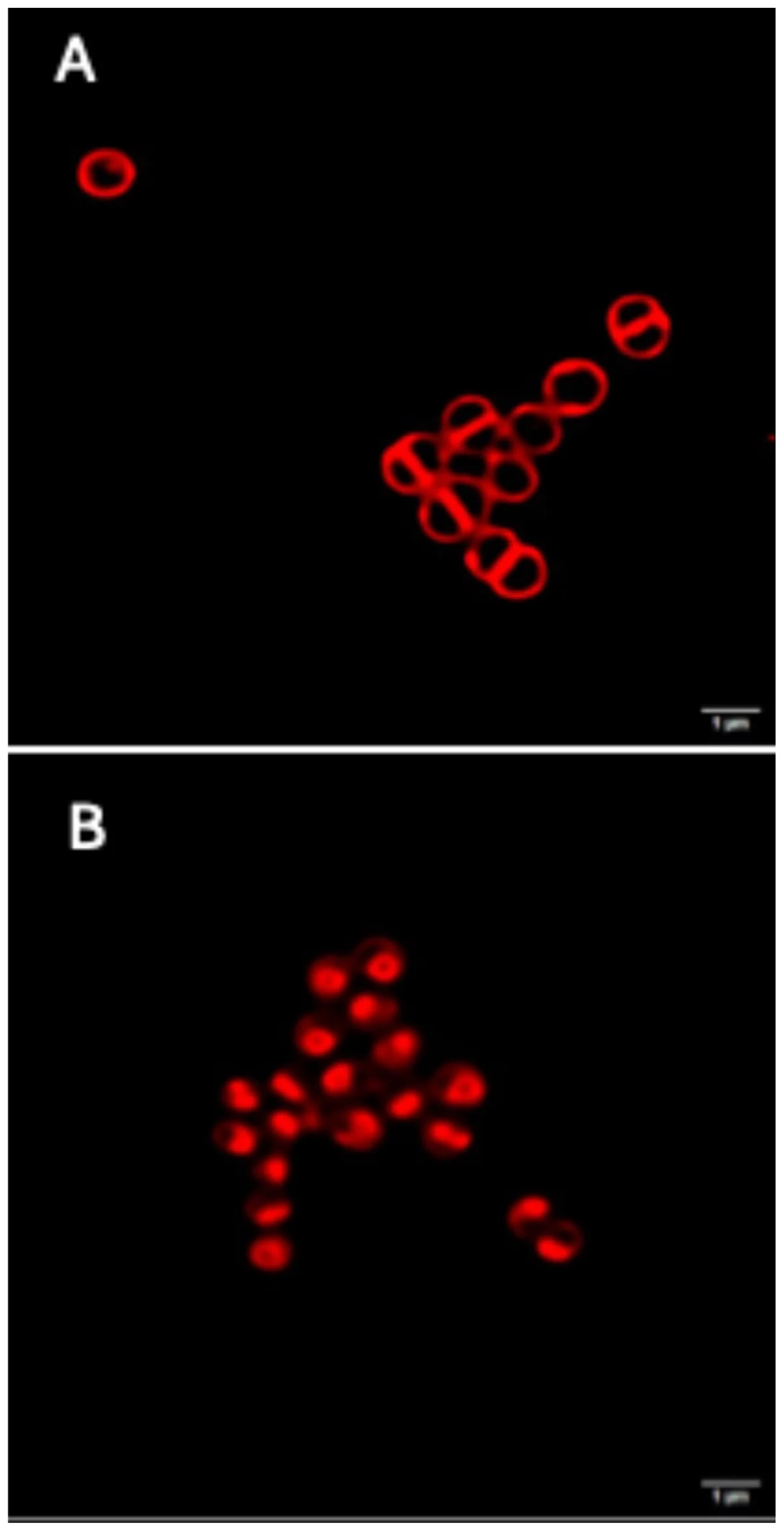
Confocal microscopy images of *S. aureus* Newman (A) control and (B) after 90’ DBCO-CD + LED treatment stained with FM4-64FX (red) membrane dye.

To assess how DBCO-CD interacts with the *S. aureus* bacterial membrane, cells incubated with either COOH-CD or DBCO-CD, with or without LED irradiation for 90 min and then stained with membrane dye FM 4-64FX (red) were imaged using confocal microscopy. Across most conditions, no noticeable changes in membrane morphology were observed. The only clear changes occurred for DBCO-CD-treated *S. aureus* after LED irradiation (Fig. 4), where the membrane appeared visibly damaged, as evidenced by a transition from uniform, peripheral to more localised dye distribution (see more images in SI).

**Fig. 5:**
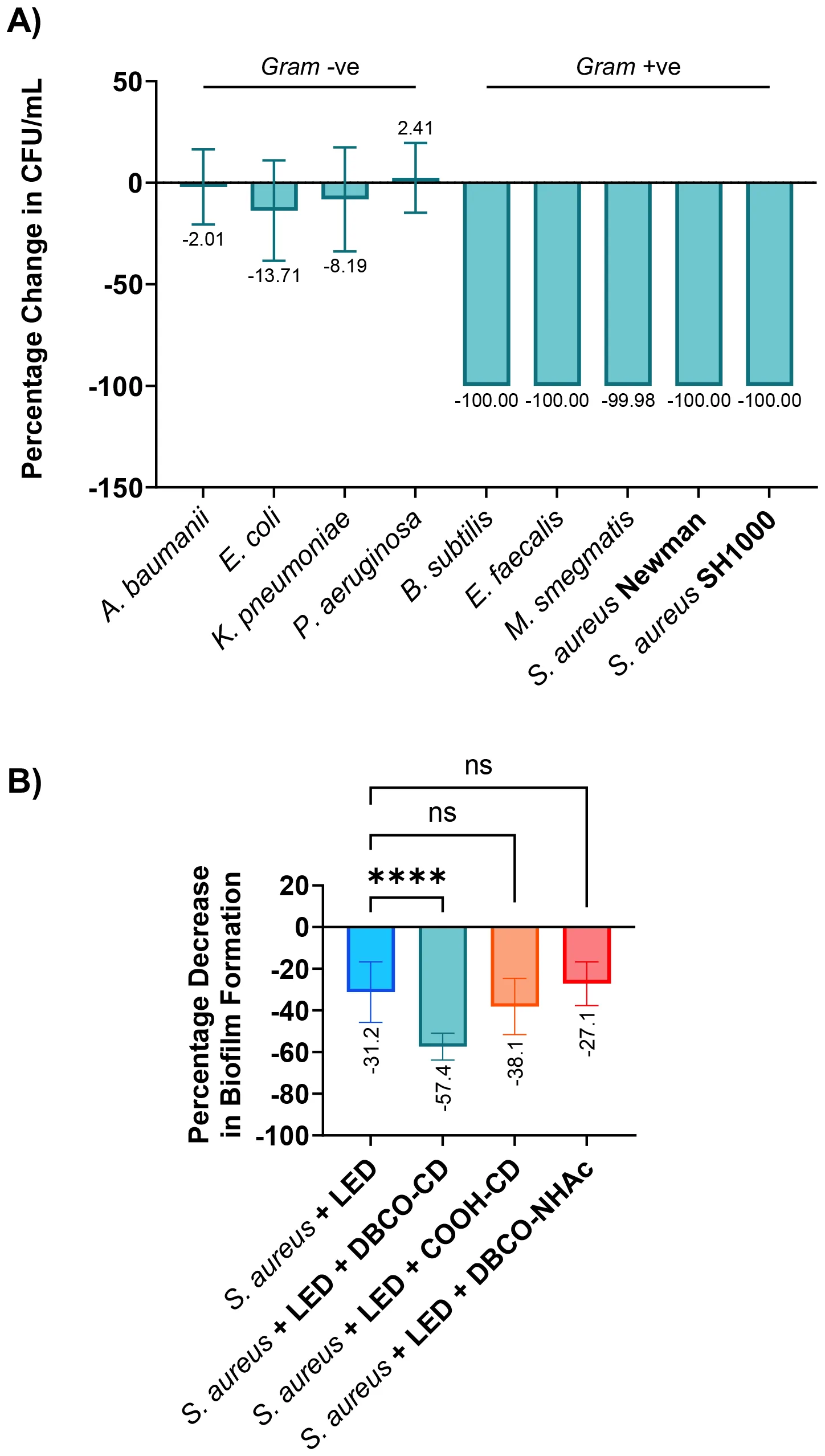
(A) Percentage change in viable cell counts of a range of bacterial species exposed to DBCO-CD (200 μg mL^−1^) after 90 min irradiation with 395–405 nm LED. (B) Percentage change in biomass of mature *S. aureus* SH1000 biofilms exposed to COOH-CD, DBCO-CD and DBCO-NHAc with 90 min. LED irradiation compared to an untreated, unirradiated control. Statistical significance assessed *via* two-way ANOVA. (*p* > 0.05 = ns, *p* < 0.0001 = ****). Error bars indicate ± standard deviation of replicates.

Intrigued by the selectivity towards *S. aureus* over *E. coli* exhibited by DBCO-CD, we evaluated its effect across a broader panel of bacterial species using a slightly modified protocol from our group.^7^*S. aureus* Newman and SH1000, *M. smegmatis* mc^2^155, *E. faecalis* NCTC 12201 and *B. subtilis* PY79 were chosen as Gram-positive examples, while *E. coli* BW25113, *P. aeruginosa* PA01, *K. pneumoniae* NCTC 5055 and *A. baumannii* ATCC 19606 were included as Gram-negative representatives. As previously observed for *E. coli* and *S. aureus*, LED irradiation alone, as well as treatment with DBCO-CD in the dark, showed no significant reduction in cell viability compared to untreated controls (see SI) for all species tested. Upon LED irradiation, samples treated with DBCO-CD showed substantial antibacterial activity towards all Gram-positive species tested, yielding a >99.98% reduction in viable cell count. In contrast, no statistically significant effect was observed on any Gram-negative species, consistent with previous observations of Gram-negative bacteria being more resistant towards ROS (Fig. 5A).^15^

Given the pronounced cytotoxicity of LED-irradiated DBCO-CD toward planktonic bacteria, we next examined the effects of COOH-CD, DBCO-CD and DBCO-NHAc on *S. aureus* SH1000 biofilms. Pre-formed biofilms were incubated for 90 minutes with PBS only, or solutions of each compound of interest in PBS, either under LED irradiation or in the dark. A small reduction in biofilm formation in the dark was observed when the two different nanoparticles were introduced, with a decrease of 19% for DBCO-CD and 12% for COOH-CD. This is potentially attributable to abrasion resulting from exposure to the nanomaterials.^61^ A more modest reduction (7%) was observed upon treatment with DBCO-NHAc. Following 90 minutes LED (395–405 nm) irradiation, partial biofilm disruption was detected in all tested conditions (Fig. 5B). LED treatment alone produced a 31% reduction in biofilm biomass, indicating that irradiation contributes to *S. aureus* SH1000 biofilm destabilization. A comparable effect (−27%) was observed for DBCO-NHAc + LED, while the introduction of COOH-CD with irradiation enhanced this to −38%. The most pronounced disruption was observed for DBCO-CD-treated biofilms, which exhibited a 57% reduction after irradiation. Overall, the substantial biofilm disruption achieved by DBCO-CD under LED irradiation appears to arise from a synergistic combination of nanoparticle-mediated mechanical effects and DBCO-driven photodynamic activity. It is possible that abrasion associated with nanoparticles may enhance penetration of DBCO functional groups into the biofilm matrix. Furthermore, as demonstrated above, irradiation of DBCO-CD promotes ROS generation, which is well known to contribute to biofilm destabilisation and dispersal.^62^

## Conclusions

In summary, we have demonstrated that surface functionalisation of carbon dots with a strained dibenzocyclooctyne (DBCO) motif transforms otherwise inactive fluorescent nanoparticles into potent and highly selective photodynamic antibacterial agents. DBCO-CD exhibited strong light-dependent cytotoxicity against Gram-positive bacteria, achieving >99.9% killing within 90 minutes of 395–405 nm LED irradiation, while remaining inactive without irradiation and towards Gram-negative species. The intact strained alkyne is essential for activity, as conversion to the corresponding triazole or use of the small-molecule DBCO analogue significantly diminished antibacterial efficacy. ROS-scavenging experiments implicated H_2_O_2_-mediated oxidative stress as a principal contributor to bacterial inactivation, while sub-cellular fluctuation imaging revealed a profound collapse of intracellular dynamics consistent with rapid metabolic failure. Importantly, DBCO-CD also disrupted established *S. aureus* biofilms, achieving a 57% reduction in biomass following irradiation. Collectively, these findings establish strained-alkyne surface engineering as a powerful and modular strategy to endow carbon dots with strong photodynamic antibacterial function. Unlike conventional APDT systems that rely on embedded organic chromophores or metal-based sensitizers, this approach employs surface chemistry to introduce photoactivity, offering a metal-free, synthetically accessible, and tuneable platform. The ability to combine bioorthogonal click functionality with intrinsic photodynamic activity opens new opportunities for targeted antimicrobial design and the development of next-generation antibiofilm materials to address the growing challenge of antimicrobial resistance.

## Author contributions

CS: chemical synthesis and characterization. CS, AE and YT investigation, methodology and formal analysis. CH: data curation and statistical analysis. MCG, MA and JS: conceptualization, project administration, direct lab supervision, funding acquisition, formal analysis and data validation. All authors contributed to the writing – review & editing.

## Conflicts of interest

There are no conflicts to declare.

## Supplementary Material

NR-018-D6NR01923D-s001

## Data Availability

The data supporting this article have been included as part of the supplementary information (SI). Supplementary information: synthetic protocols and characterization data for all probes, including all bacteria screening and biofilm studies. See DOI: https://doi.org/10.1039/d6nr01923d.
