# Supplementary material for: Strained alkyne functionalization of carbon dots enables selective photodynamic killing of Gram-positive bacteria
Source: Nanoscale. 2026 Aug 7;18(35):19157–64. doi: 10.1039/d6nr01923d (PMC13491393; doi:10.1039/d6nr01923d)
Supplement: Supplementary file 1 [file NR-018-D6NR01923D-s001.pdf]

Supplementary Information (SI) for Nanoscale.
This journal is © The Royal Society of Chemistry 2026

Supporting Information

Strained Alkyne Functionalization of Carbon Dots Enables Selective Photodynamic Killing of Gram-Positive Bacteria

Cristina M. Santi[a], *Arthur Eley*[b], *Yuiko Takebayashi*[c], *Calum S. Haydon*[a], *James Spencer*[c]*, *M. Antognozzi*[b]*, *M. Carmen Galan*[a]*

Materials and Methods

All the chemicals were purchased from Sigma Aldrich or Fisher Scientific and used without further purification. NMR spectra were recorded in the appropriate deuterated solvent (purchased from Sigma Aldrich) at 400 MHz on a Brucker or 600 MHz on a Brucker cryoprobe spectroscope. The chemical shifts were referenced to the residual solvent peak and reported in ppm.

S. aureus strain Newman and *E. faecalis* NCTC 12201 were gifts from Jim Spencer, *S. aureus* SH1000 from Ruth Massey and *K. pneumoniae* NCTC 5055 from Matthew B Avison (University of Bristol). *A. baumannii* ATCC 19606 was a gift from Benjamin A Evans (University of East Anglia). *E. coli* BW25113 was purchased from the *E. coli* Genetic Stock Center (Yale CSC, New Haven, United States). *B. subtilis* PY79 was purchased from the Bacillus Genetic Stock Center (The Ohio State University, United States). *P. aeruginosa* PA01 was obtained from the *Pseudomonas* Genetic Stock Centre (East Carolina University, United States). *M. smegmatis* mc^{2}155 was purchased from atcc.org. All bacteria suspensions' OD_{600} were measured with Ultrospec 2100 pro (Amersham Biosciences) UV/Visible Spectrophotometer. Crystal violet fluorescence was measured on a CLARIOstar (BMG Labtech) plate reader, using Costar 96-well, flat bottom plates.

Synthesis

COOH-CD: COOH-CD have been synthesized according to our previous work.[1] Briefly, 1.0 g of citric acid (5.2 mmol), 0.79 g of L-cysteine (6.50 mmol) and 4.92 g of urea (83.11 mmol) were dissolved in 20 mL distilled H_2O in a 250 mL conical flask and stirred for 20'. The solution was placed in a domestic microwave at 600W and reacted for 3'. The residue was resuspended in 20 mL distilled H_2O and purified via centrifuge-filtration using

Vivaspin concentrators (MWCO 10kDa, 8500 rpm, 30 min). The filtrate was dialyzed for 2 days in a 100-500Da dialysis bag, changing water 4 times. The residue was freeze-dried to yield an amber powder of **COOH-CD** (0.90 g).

All characterization data were in agreement to previously reported data.[1]

DBCO-CD: COOH-CD (20 mg) were suspended in 1 mL distilled H_2O in a 2 mL Eppendorf tube. Carbonyl diimidazole (CDI, 40 mg, 0.25 mmol) was added, and the mix was sonicated for 15'. Dibenzo cyclooctyne-amine (10 mg, 0.036 mmol) was added, and the mixture was sonicated for further 2h. The crude was purified via dialysis 100-500Da for 2 days, changing water 4 times. The solution was freeze-dried to yield a yellowish powder of **DBCO-CD** (18 mg). The amount of DBCO present on **DBCO-CD**, quantified by ^{1}H NMR (internal standard: maleic acid), was 0.50 μmol/mg.

Gal-CD: DBCO-CD (8 mg) were suspended in 0.3 mL distilled H_2O in a 0.5 mL Eppendorf tube. 1-O-(3-azidopropyl)-β-D-galactopyranoside (2.6 mg, 0.010 mmol) was added, and the mix was sonicated for 2 h. The crude was purified via dialysis 100-500 Da for 1 day, changing water 2 times. The solution was freeze-dried to yield an amber powder of **Gal-CD** (7 mg).

Aza-dibenzocyclooctyne-amide (DBCO-NHAc): DBCO-amine (10 mg, 0.036 mmol) was dissolved in DCM (200 μL) and DIPEA (9.5 μL, 1.5 eq.). Ac_2O (4.1 μL, 1.2 eq.) was added, and the mixture was stirred for 2 h at RT. The solvent was removed in vacuum, and the crude was purified by flash chromatography (DCM + 1% MeOH). **DBCO-NHAc** (9.0mg) was isolated in 78% yield. ^{1}H NMR (400 MHz, $CDCl_3$) δ: 7.67 (d, J = 7.8Hz, 1H), 7.42-7.27 (m, 7H), 5.96 (s, 1H), 5.13 (d, J = 13.8Hz, 1H), 3.70 (d, J =13.8 Hz, 1H), 3.34 (m, 1H), 3.19 (m, 1H), 2.44 (m, 1H), 1.94 (m, 1H), 1.80 (s, 3H). The spectroscopic data were in agreement with literature.[2]

CD-probes characterization

COOH-CD ^{1}H NMR (500 MHz, Deuterium Oxide)[1]

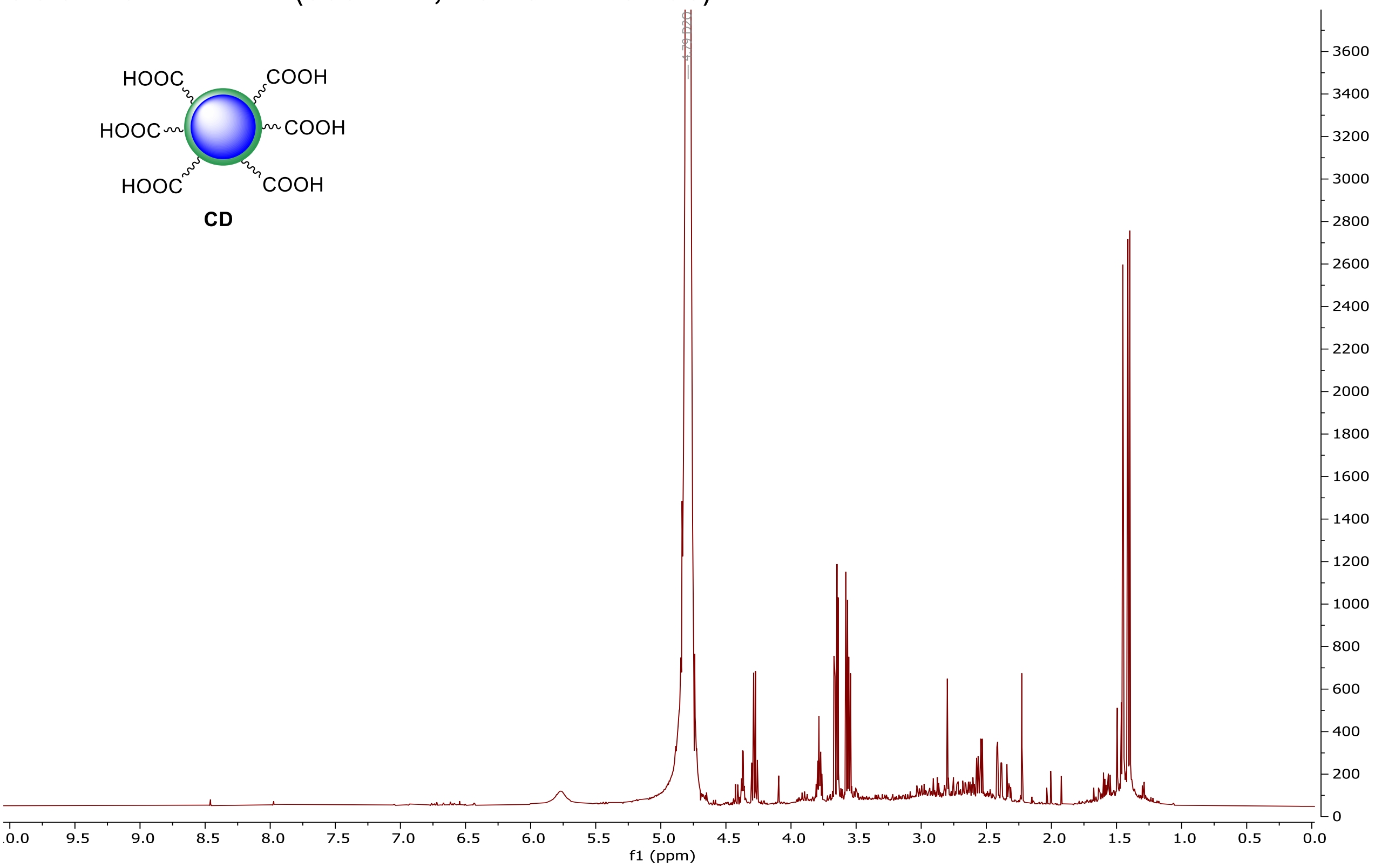

COOH-CD Zeta potential

Results

			Mean (mV)	Area (%)	St Dev (mV)
**Zeta Potential (mV):**	-8.32	**Peak 1:**	-8.04	100.0	7.91
**Zeta Deviation (mV):**	7.39	**Peak 2:**	0.00	0.0	0.00
**Conductivity (mS/cm):**	0.123	**Peak 3:**	0.00	0.0	0.00
**Result quality :**	Good				

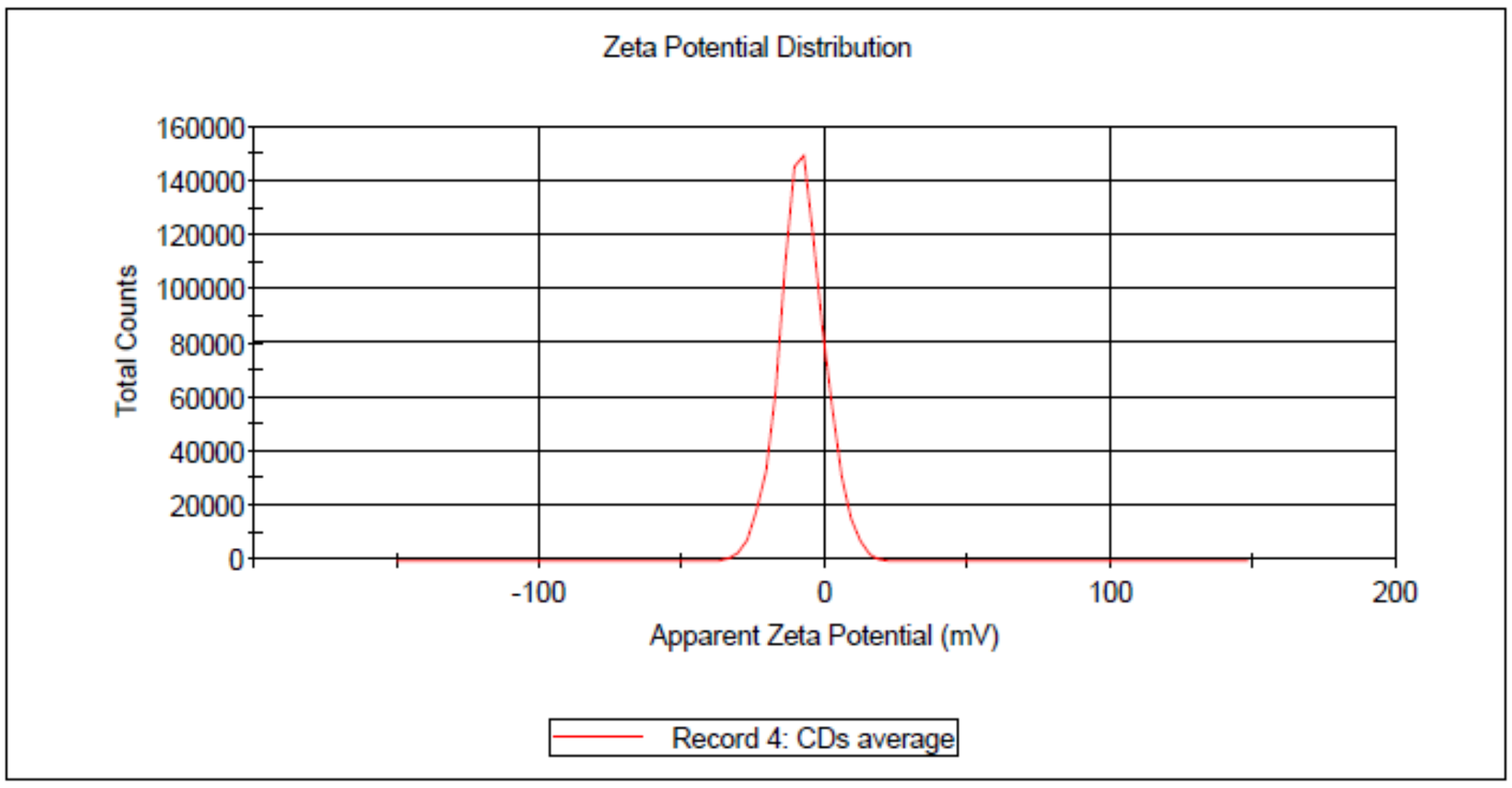

DBCO-CD ^{1}H NMR (500 MHz, Deuterium Oxide)

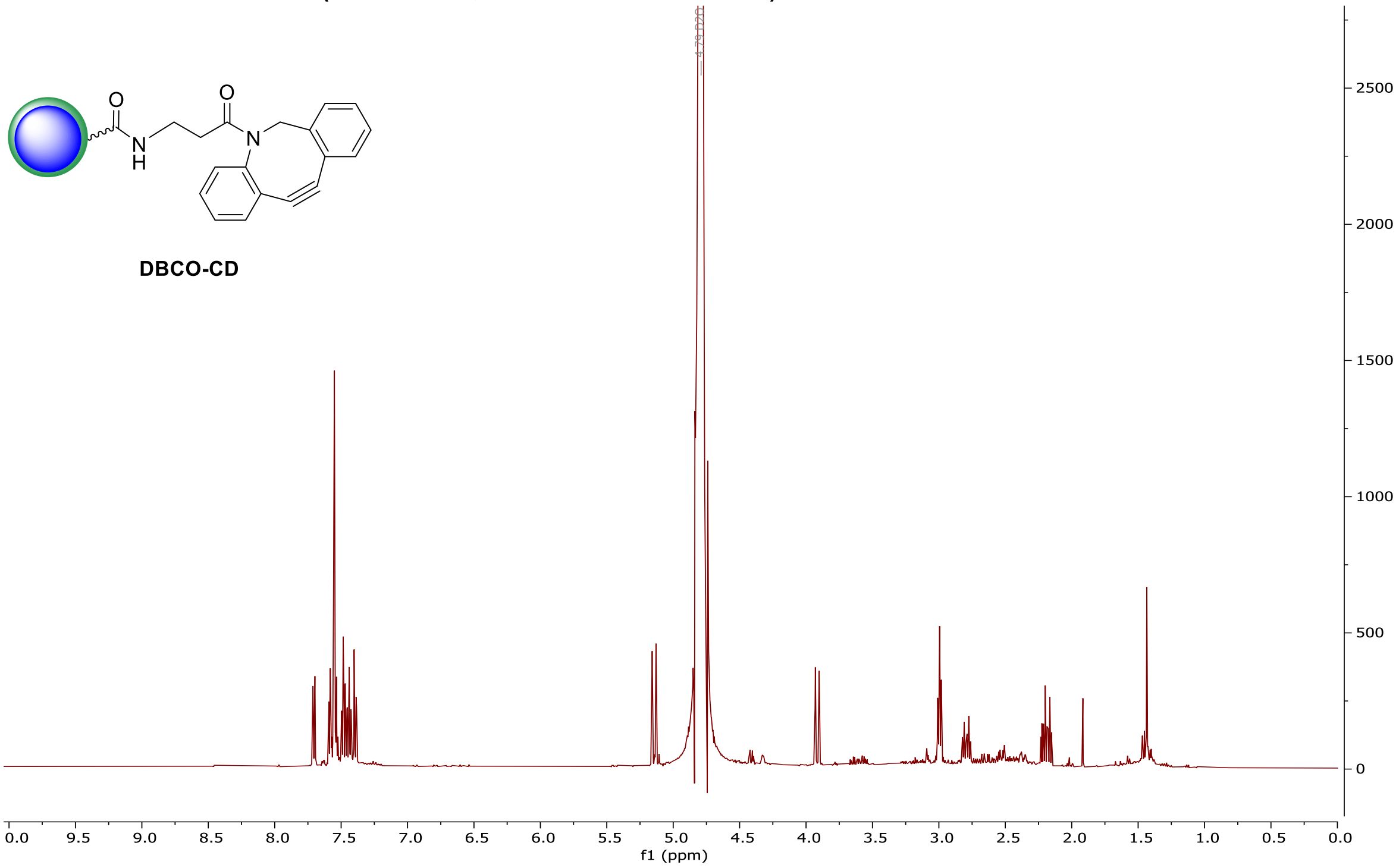

DBCO-CD ^{13}C NMR (126 MHz, D_2O)

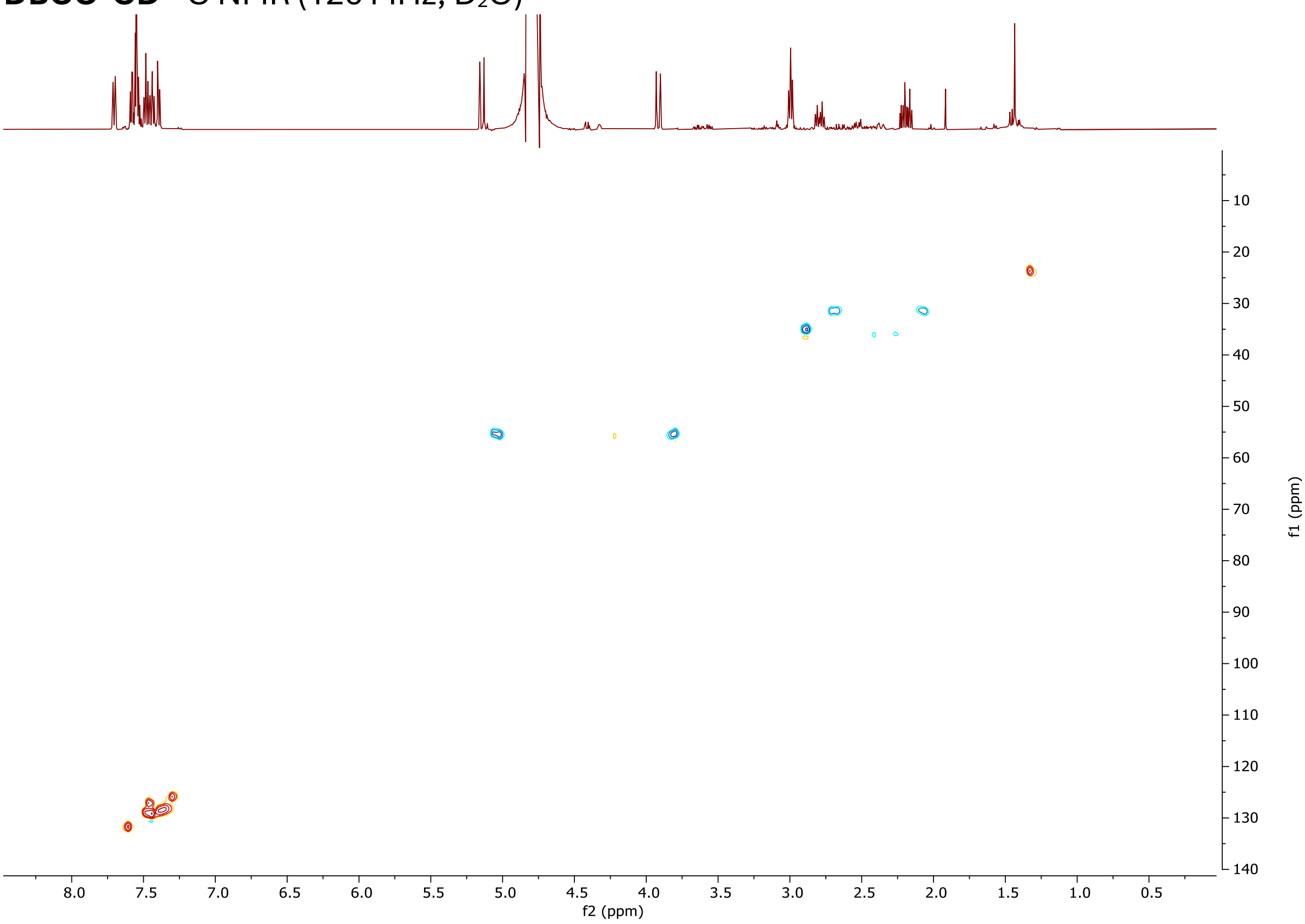

DBCO-CD Zeta potential

Results

			Mean (mV)	Area (%)	St Dev (mV)
**Zeta Potential (mV):**	-9.05	**Peak 1:**	-9.10	100.0	5.19
**Zeta Deviation (mV):**	4.69	**Peak 2:**	0.00	0.0	0.00
**Conductivity (mS/cm):**	0.230	**Peak 3:**	0.00	0.0	0.00
**Result quality :**	Good				

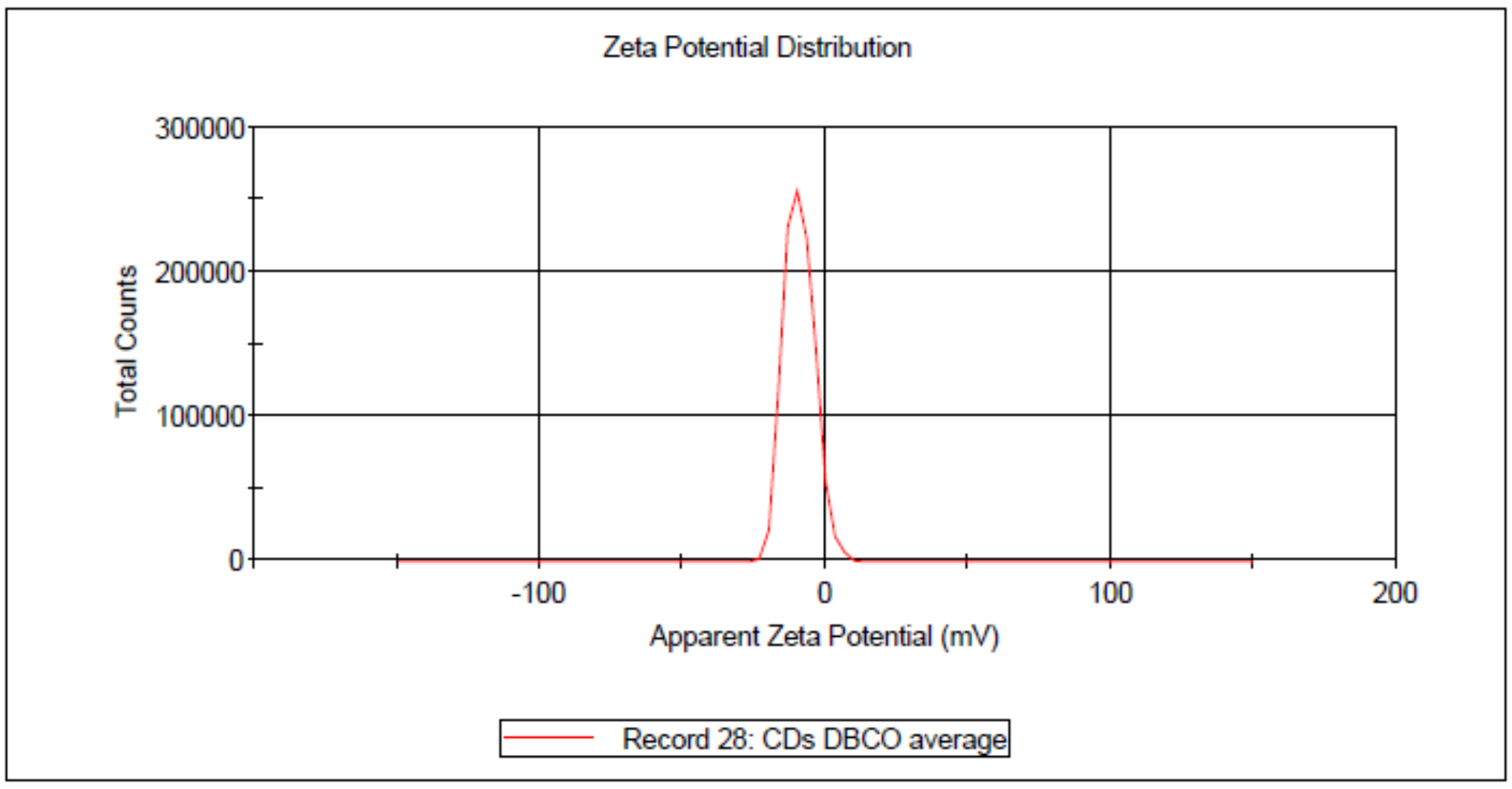

DBCO quantification in CD-DBCO samples

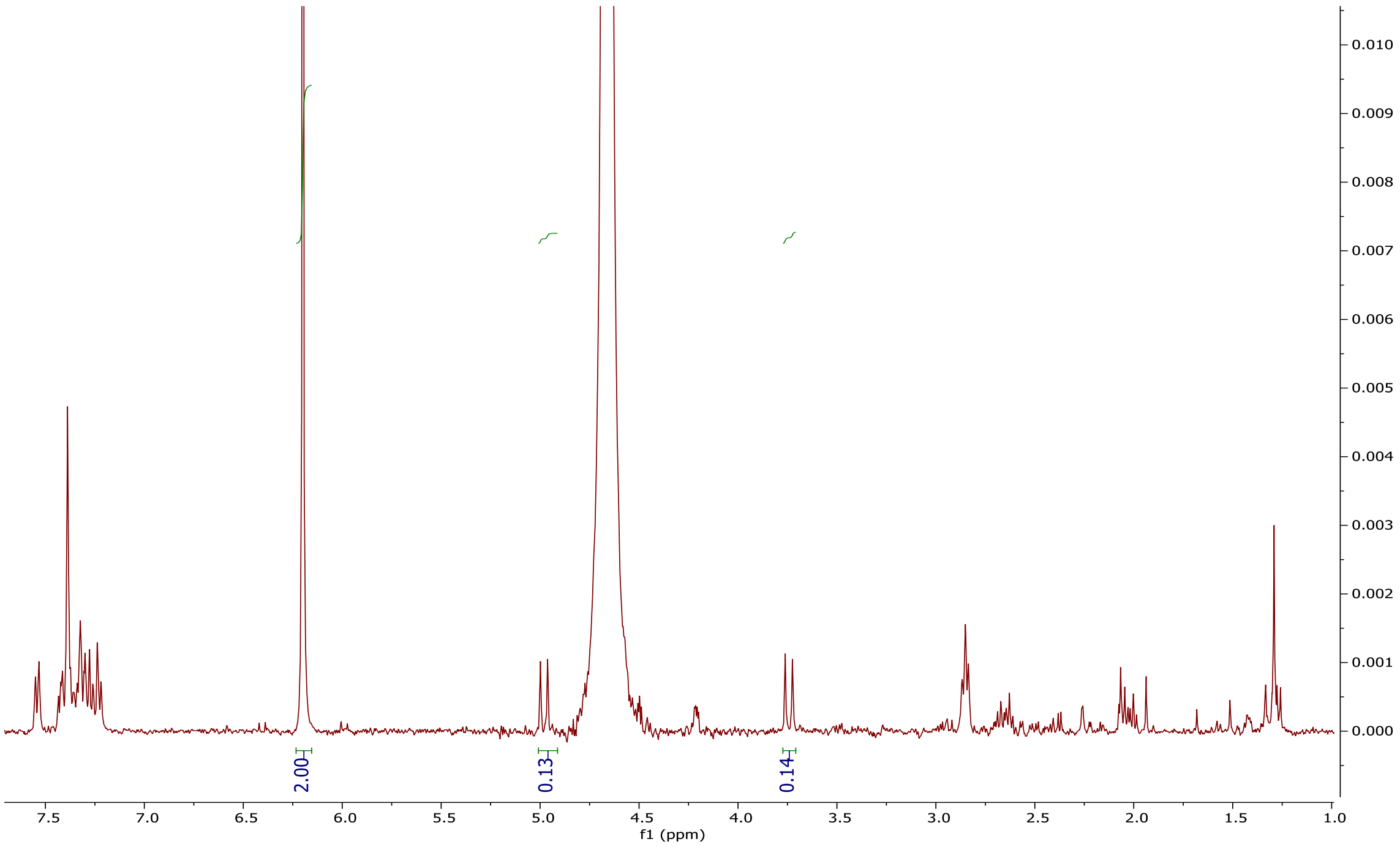

[maleic acid] in the NMR sample =	1.3mg/ml	NMR ratio ($n_{maleic\ acid}/n_{DBCO}$) =	1 : 0.135
[**CD-DBCO**] in the NMR sample =	3.0mg/ml	**DBCO in CDs =**	0.50µmol/mg

^{1}H NMR spectrum of **CD-DBCO** + maleic acid (internal standard) and **DBCO** quantification.

Gal-CD ^{1}H NMR (600 MHz, D_2O)

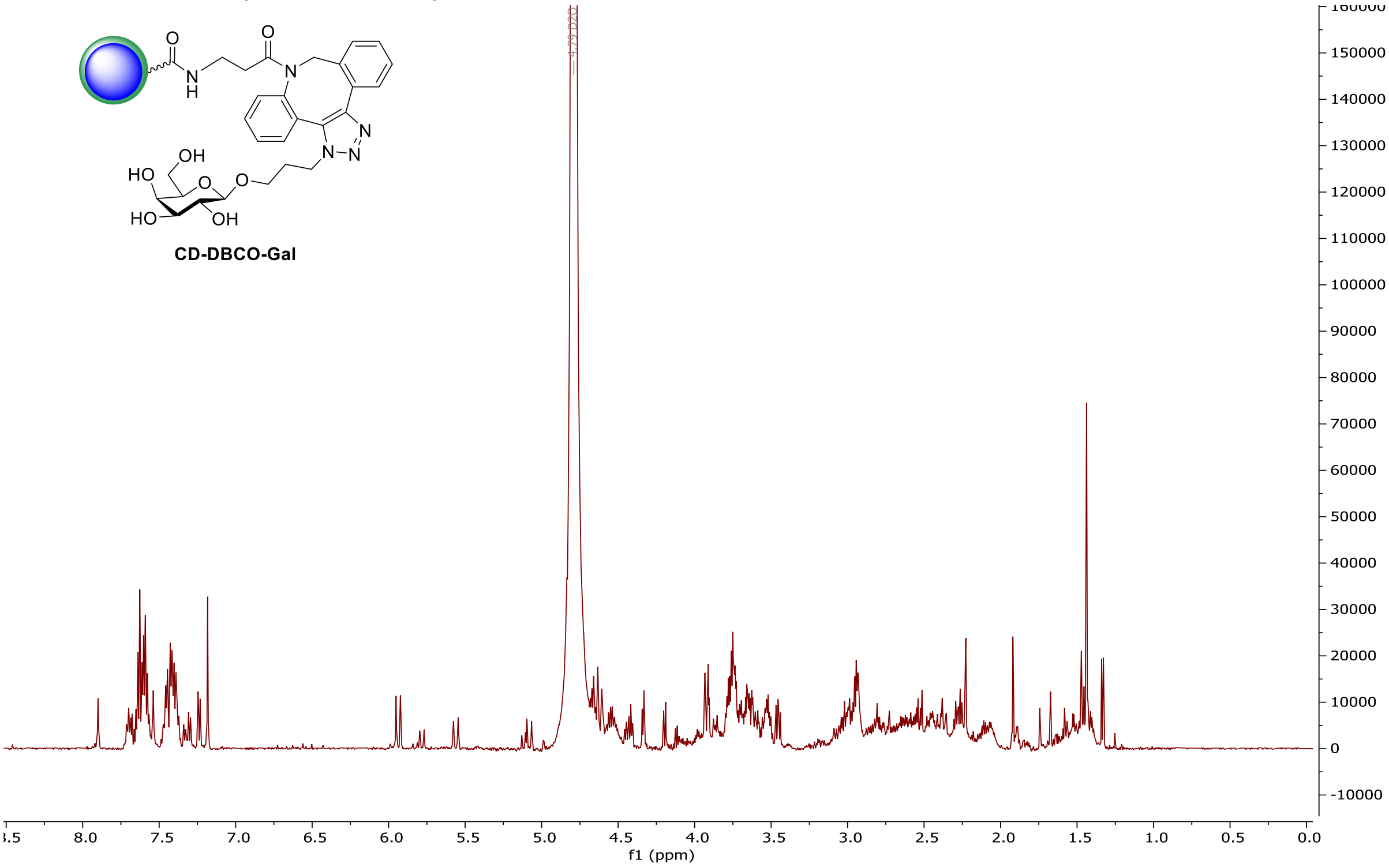

Gal-CD HSQC NMR (600 MHz, D_2O)

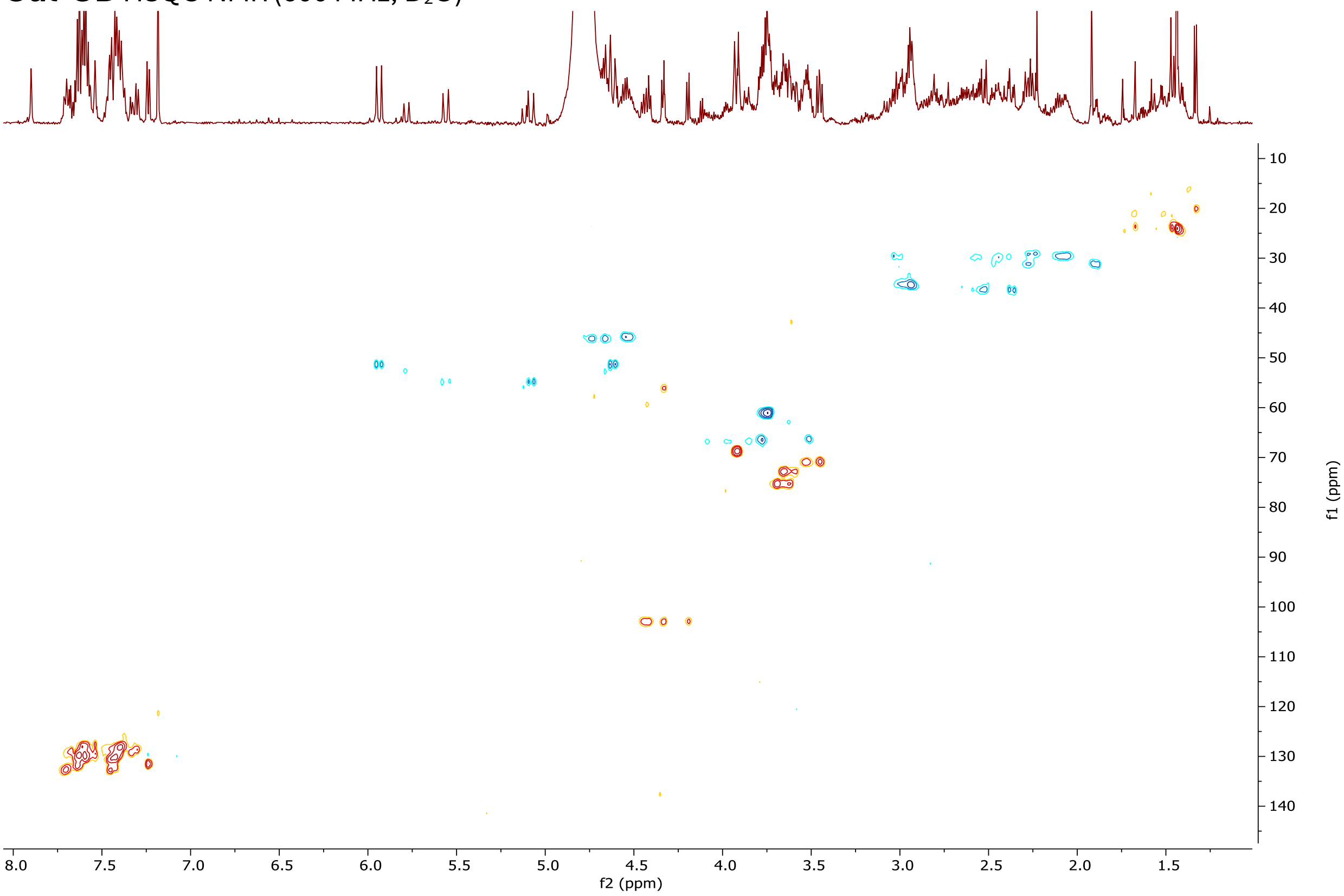

Gal-CD Zeta potential

Results

			Mean (mV)	Area (%)	St Dev (mV)
Zeta Potential (mV):	-8.43	Peak 1:	-8.45	100.0	3.56
Zeta Deviation (mV):	3.39	Peak 2:	0.00	0.0	0.00
Conductivity (mS/cm):	0.276	Peak 3:	0.00	0.0	0.00
Result quality :	Good				

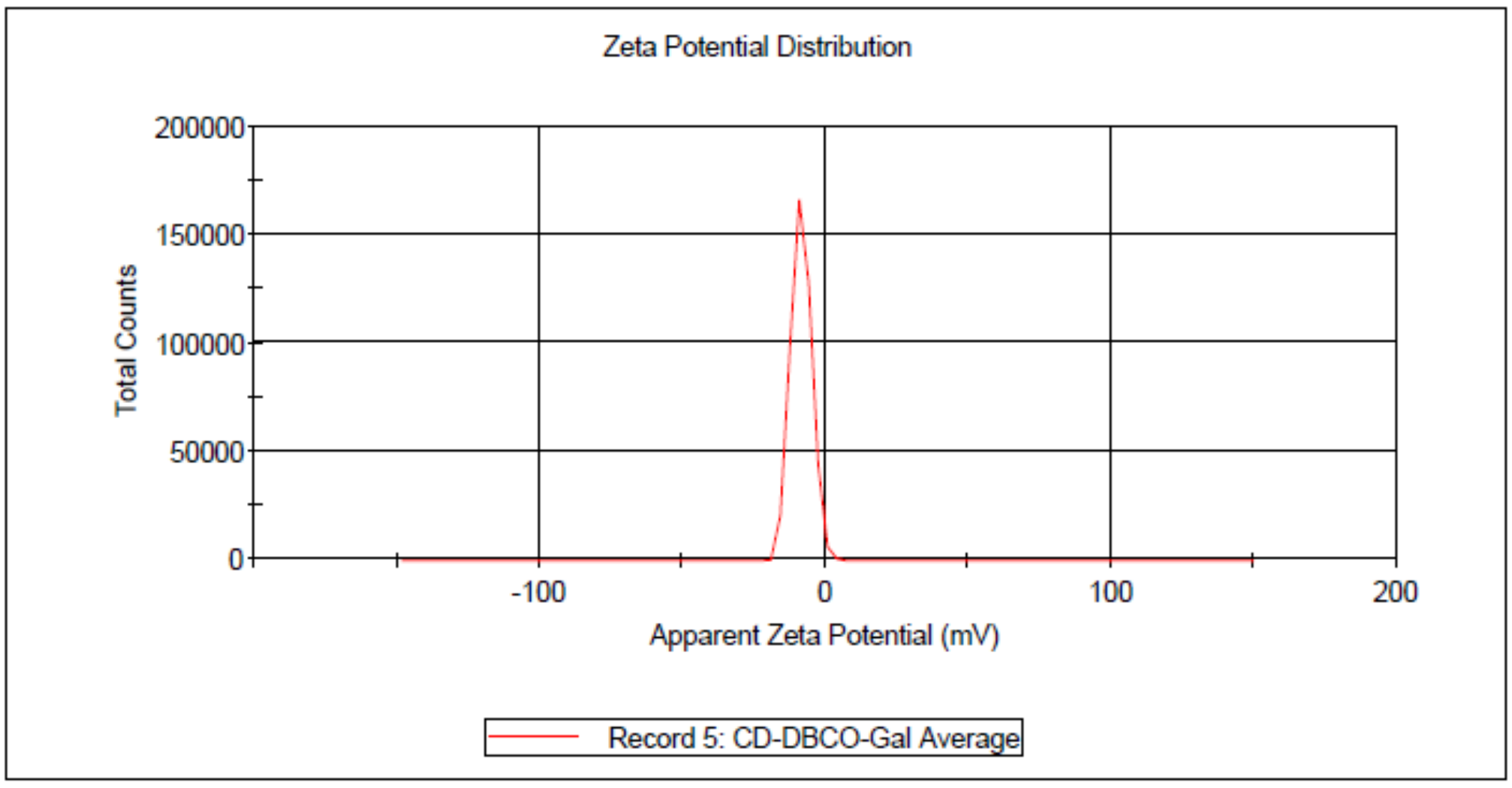

Bacterial Culture Protocol: *M. smegmatis* mc^{2}155 was grown in 10 mL Middlebrook 7H9 (LB) broth containing 0.05% Tween 80 for 24 h, 180 rpm at 37 °C. All other bacteria were grown in 10 mL LB broth for 16-18 h, 180 rpm at 37 °C.

Bacterial Killing Assay: 96 µL of bacteria (OD600 = 0.8-1.0) in sterile PBS was incubated with 4 µL of a 5 mg/mL suspension of **COOH-CD**, **DBCO-CD** or **Gal-CD in** sterile water (to a final carbon dot concentration of 200 µg/mL and a DBCO concentration of 0.100 µmol/ml in **DBCO-CD**), with 4 µL of a 818 µg/mL **DBCO-NHAc** solution (0.100 µmol/ml final concentration) or with 4 µL sterile water (control). When required, a 100 mM solution of sodium pyruvate in PBS was used instead of PBS. The samples were incubated with and without LED light irradiation (wavelength= 395-405 nm) for 90 min. Serial dilutions with sterile PBS (up to 10^8) were performed and dilutions ranging from 10^1 and 10^8 were spread on LB agar plates and incubated at 37 °C overnight (3 days for *M. smegmatis*). The number of colonies were then counted (for plates with 30<number of colonies>300), and original bacterial concentration calculated. Each experiment has been repeated in biological and technical triplicate. Bacterial killing effectiveness was reported as the

percentage decrease in CFU/mL relative to the CFU/mL of an un-irradiated bacteria only control sample (% decrease = [(Treated CFU/mL)-(Untreated CFU/mL)]/(Untreated CFU/mL)).

Fluorescent Labelling of Cells by CDs

S. aureus Newman overnight cultures were diluted in PBS to OD_{600} = 1.0 (~ 10^9 CFU/mL). To 300 µL bacterial suspension in PBS was added stock solutions of **COOH-CD** or **DBCO-CD** to final concentrations of 400 µg/mL. These were incubated in darkness with gentle agitation for 1 h. The cells were then centrifuged at 3000 xg for 5 minutes, the supernatant removed, and the pellet washed with PBS to remove any unbound CDs, this procedure was repeated twice. 3 × 100 µL of the resultant washed bacterial suspension was aliquoted into a 96 well plate, and the fluorescence intensities for each sample were recorded at excitation/emission: 340/450 nm and 391/499 nm (Fig. S1).

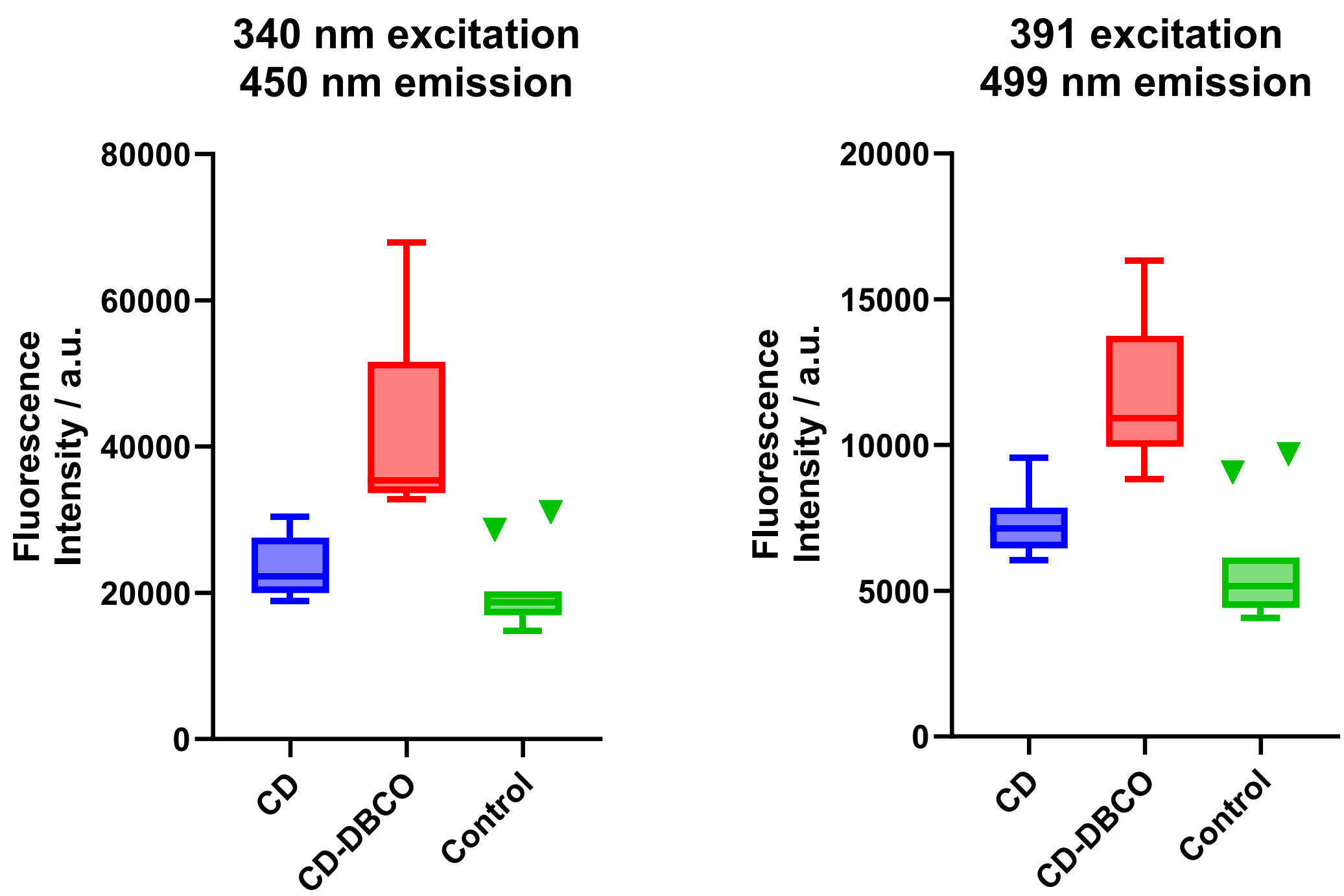

Figure S1. Fluorescence emission intensity of *S. aureus* SH1000 cells labelled with **COOH-CD** and **DBCO-CD**, compared to background fluorescence of an unlabelled *S. aureus* control.

Confocal Imaging: 240 µL of *S. aureus* (OD_{600}= 0.8-1.0) in sterile PBS was incubated with 10 µL of a 5 mg/mL suspension of **DBCO-CD** or **COOH-CD** (to a final carbon dot concentration of 200 µg/mL) or with 10 µL sterile water (control). The samples were incubated with LED light irradiation (wavelength= 395-405 nm) or in dark (No LED control) for 90 min. Subsequently, the suspensions were centrifuged at 5000 xg for 5 minutes and the cell pellets washed twice with PBS before adding FM4-64FX (ThermoFisher) to a final concentration of 1 ug/mL and incubating for 1 min at room temperature. The samples were then centrifuged at 5000 xg for 30 seconds, aspirated to 50 ul and the cell pellets resuspended. 5 ul sample was transferred onto a 1.2% w/v agarose pad and mounted with a coverslip. All images were acquired on the Leica TCS SP8 system attached to a Leica DMi8 inverted microscope (Leica Microsystems) using a 63x/1.4 HC PL APO CS2 oil immersion objective. The samples were excited using a DPSS 561 laser for detecting FM4-64FX (emission range 570-650 nm) and an argon laser for CD-DBCO (emission range 495-550 nm) and the acquired images deconvoluted using Leica's Lightning process.

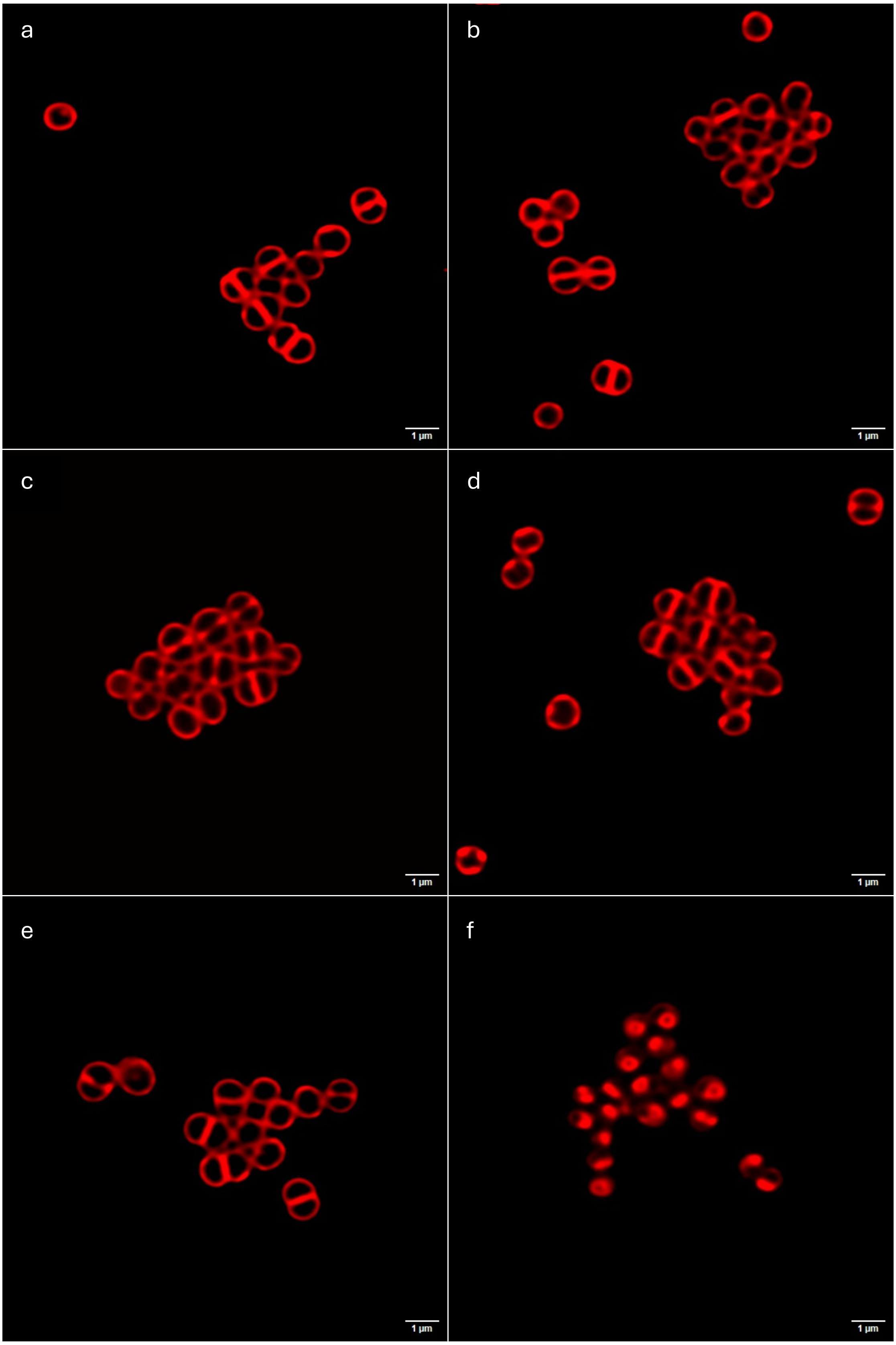

Figure S2. Confocal microscopy images of *S. aureus* Newman a) control and after b) 90' LED, c) **CD**, d) **CD** + LED, e) **CD-DBCO**, f) **CD-DBCO** + LED treatment stained with FM4-64FX (red) membrane dye.

Temperature control measurements: 240 µL of bacterial suspensions in PBS were mixed with either 10 µL of **COOH-CD** or **DBCO-CD** 5mg/mL suspension in sterile water (200 µg/mL final concentration) or 10 µL sterile water (control) in centrifuge tubes. The tubes were irradiated with LED (λ=395-405nm) and temperature was recorded after 0, 1, 3, 5, 7, 15, 30 and 90 minutes. A Thermocouple (type K from Fisher Scientific) was used to record temperature to 1 decimal place. The experiment was repeated in triplicate (Figure S3).

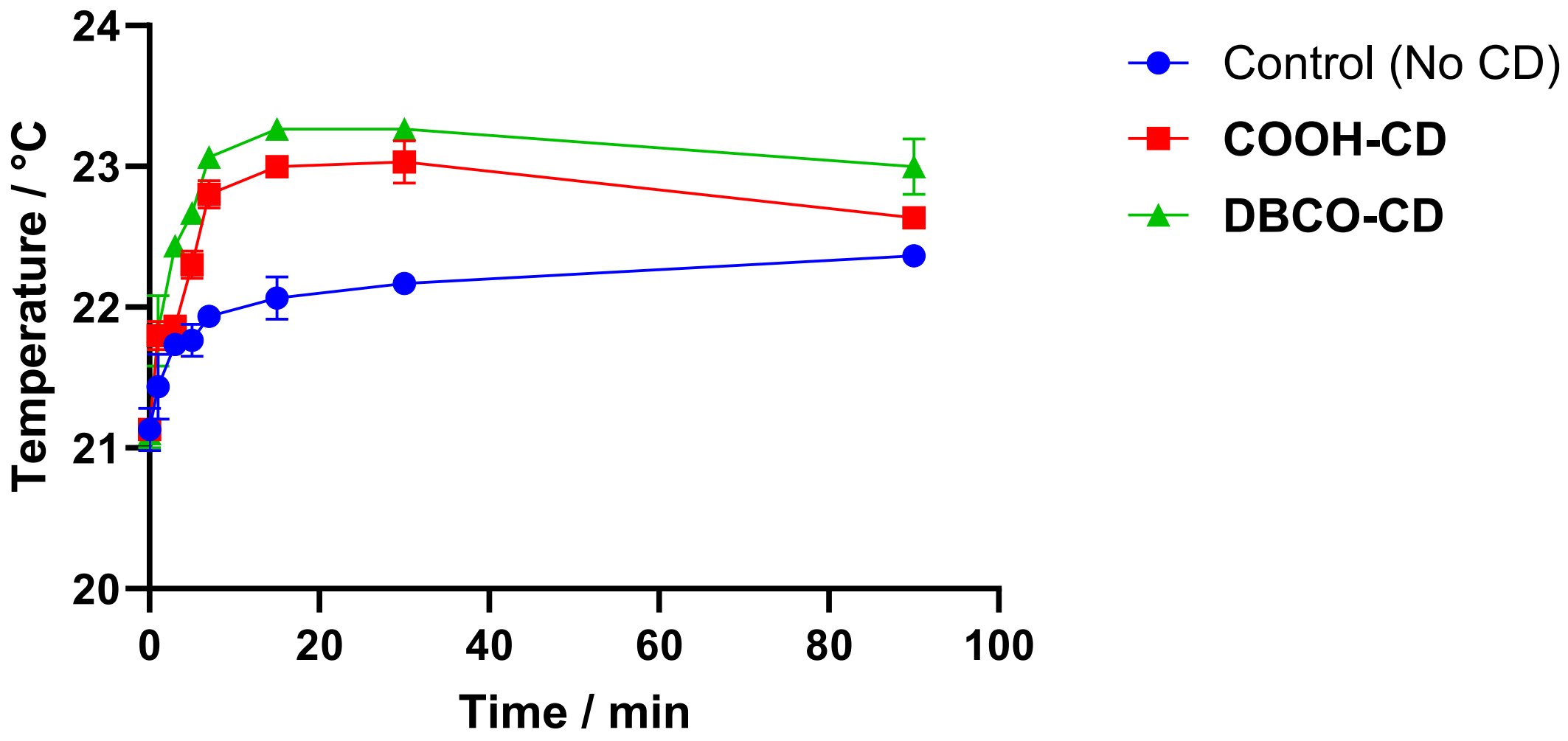

Figure S3: Temperature of *S. aureus* and **COOH-/DBCO-CD** solutions (200 µg/mL) under 395-405nm LED irradiation at timepoints between 0-90 mins.

Biofilm Disruption Assay: *S. aureus* SH1000 liquid overnights were diluted in LB broth supplemented with 1% glucose to a final concentration of 1 x 10^6 CFU/mL. 100 µL of bacterial suspension and the blank (LB+ glucose only) were aliquoted in a 96-wells plate (24 wells each), ensuring 8 technical replicates per condition. The plate was incubated at 37 °C for 24 h with 5% CO_2 to promote biofilm formation. After this time, the broth was removed, and the wells were rinsed twice with 100 mL of sterile PBS to remove planktonic cells. Then, the wells were air-dried at 37 °C for 1 h. After that, the wells were filled with 100 µL of 200 µg/ml **COOH-CD** or **DBCO-CD** or 0.115 µmol/mL **DBCO-NHAc** in PBS or PBS as control. The plate was incubated at 37 °C for 24 h. After this time, the wells were emptied, rinsed twice with 100 mL of sterile PBS and air-dried at 37 °C for 1 h. 100 µL of 0.1 % crystal violet (CV) solution was added to each well and, after 5 min, the CV solution was removed, and the wells rinsed twice with 100 µL PBS. The plate was air-dried for 1 h

and the stained cells were solubilized in 100 μL of aqueous 30% acetic acid for 30 min. 20 μL of solution from each well was diluted in 200 μL of 30% acetic acid into a new plate and absorbance at 595 nm was quantified using a plate reader. The experiment was repeated in biological duplicates, eight technical replicates each. Biofilm inhibition was reported as the percentage decrease in CV absorbance at 595 nm relative to the absorbance of an un-irradiated *S. aureus* only control sample (% decrease = [(Treated CV absorbance)-(Untreated CV absorbance)]/(Untreated CV absorbance)).

SCFI Microscope design:

The SCFI system is an imaging-based method to quantify the level of activity within a single cell. The system is based on an inverted microscope, with a configuration similar to that used for total internal reflection fluorescence (TIRF) microscopy. A high-NA (1.49) objective lens is used to deliver a laser beam at a supercritical angle to the glass–sample interface, where total internal reflection generates a shallow evanescent field that illuminates the underside of the sample. The field decays over a few hundred nanometres, creating a sensitive illumination region confined to the near-surface volume of the cell.[57] Spatial variations in optical density within the cytoplasm, arising from the many different sub-cellular structures present, cause the evanescent field to be scattered by the contents of the bacterial cell. Movement of these structures changes the local optical density over time, giving rise to temporal fluctuations in scattered light intensity that SCFI measures. Scattered light is collected through the same high-NA objective lens and focused onto a CMOS detector, where intensity fluctuations are recorded at 20 fps for 20 s. A detailed description of the SCFI platform can be found in Rehnstrom et al.[57]

Theoretical principle:

The bacterial cytoplasm is glass-like, with fluidity related to the cell's metabolic activity. Metabolically active cells have a relatively fluid cytoplasm, whereas energy-depleted or dead cells have a less fluid, more glass-like cytoplasm that greatly impairs the movement of sub-cellular components. In addition, restricted movement of sub-cellular components, as a result of the glass transition, becomes more pronounced as particle size increases.[3]Consequently, energy-depleted cells are expected to exhibit local optical

density fluctuations within the cytoplasm that change over much longer timescales when compared to an equivalent healthy cell. This manifests as a reduction in both the rate constant ($1/\tau_D$) and the fluctuation amplitude (g_0) measured by SCFI.[4]

Analysis: SCFI data were recorded as a stack of 400 images at 20 fps, and analysed using temporal autocorrelation functions (ACF), a method described in detail in Krieger *et al.*, amongst others.[5, 6] For each bacterium, the video was cropped to a 20x20 pixel region of interest (ROI) within the cell. The temporal ACF was computed individually for each pixel within the ROI, and a curve was fitted to each ACF of the form:[6]

$$G(t) = G_\infty + 1/N\left(1 + \left(\frac{t}{\tau_D}\right)\right)^{-1}$$

Equation (1) gives the rate constant ($1/\tau_D$) directly, and the amplitude of fluctuation g_0 is equivalent to $1/N - G_\infty$, which can also be understood as the ratio of signal variance to the square of the signal mean intensity. [5, 6] There were 400 pixels total within the ROI for each cell, and after filtering pixels with unphysical fitted parameters (e.g., negative τ_D values or poor goodness of fit), the medians of the $1/\tau_D$ and g_0 distributions were taken forward for each cell. For each sample, 50 cells were measured to obtain a representative distribution, and each experiment was repeated in biological triplicate. To determine the *p*-value, Welch's two-sided t-test for two independent samples (assuming unequal variance) was used, with significance threshold set at $p = 0.05$.

Technical details: All bacterial suspensions were taken directly from the suspensions used in the main experiment (colony counting). Samples were immobilised on glass-bottomed petri dishes (Cellview cell culture dish, Greiner Bio-One, 627861) soaked for 10 minutes with 2mg/ml polydopamine in Tris-HCl buffer (pH 8.5). Prior to polydopamine coating, the glass substrate was rinsed with ethanol and DI water and washed with acetone before drying with a nitrogen line and plasma cleaning in pure O_2 for 6 minutes. Cell suspensions were diluted to OD_{600} ~ 0.1. Cells were contained in a flow channel, constructed on the glass substrate using biocompatible nitrile film and 3M double sided adhesive.

Microscope components: Nikon high NA (1.49) TIRF objective lens; two movable 45° rod 1x2mm mirrors from Edmund optics; Vortran Stradus 488nm laser diode, delivered by a single-mode and polarisation-maintaining optical fibre; 10mm right-angle mirror (from Edmund Optics) mounted below the aperture; tube lens that forms the image; sCMOS

sensor from a Thorlabs Quantalux 2.1 MP camera. The output from the optical fibre is collimated using a 25mm aspheric lens and focused at the back focal plane using a plano-convex lens; the beam is *s*-polarised after collimation. The evanescent field has a diameter of ~20 μm in the plane of the optical interface (1/e level). The power density used is approximately 5μW/μm^2 at the surface of the optical interface. The Thorlabs camera has a quantum efficiency of 55% at 488nm, an RMS readout noise of less than 1.5e^{-1} and a physical pixel size of 5x5μm. Given the optical magnification of this system, the effective pixel size is ~29nm per pixel. Piezo-driven inertia actuators (PIA13 from Thorlabs) provide the translational movement of the stage and motorised focus. The horizontal and vertical drift of the stage is less than 1nm during the 20 seconds of recording time.[7]

Note on Impact of Environmental Conditions on SCFI results

Significant differences were also observed when comparing control groups across experiments, attributable to day-to-day variability in environmental conditions and microscope configuration, particularly the evanescent field depth and alignment. Triplicate measurements were performed under consistent conditions within each experiment, but longer intervals between separate treatment studies likely contributed to shifts in control distributions, consistent with the high sensitivity of the SCFI system.

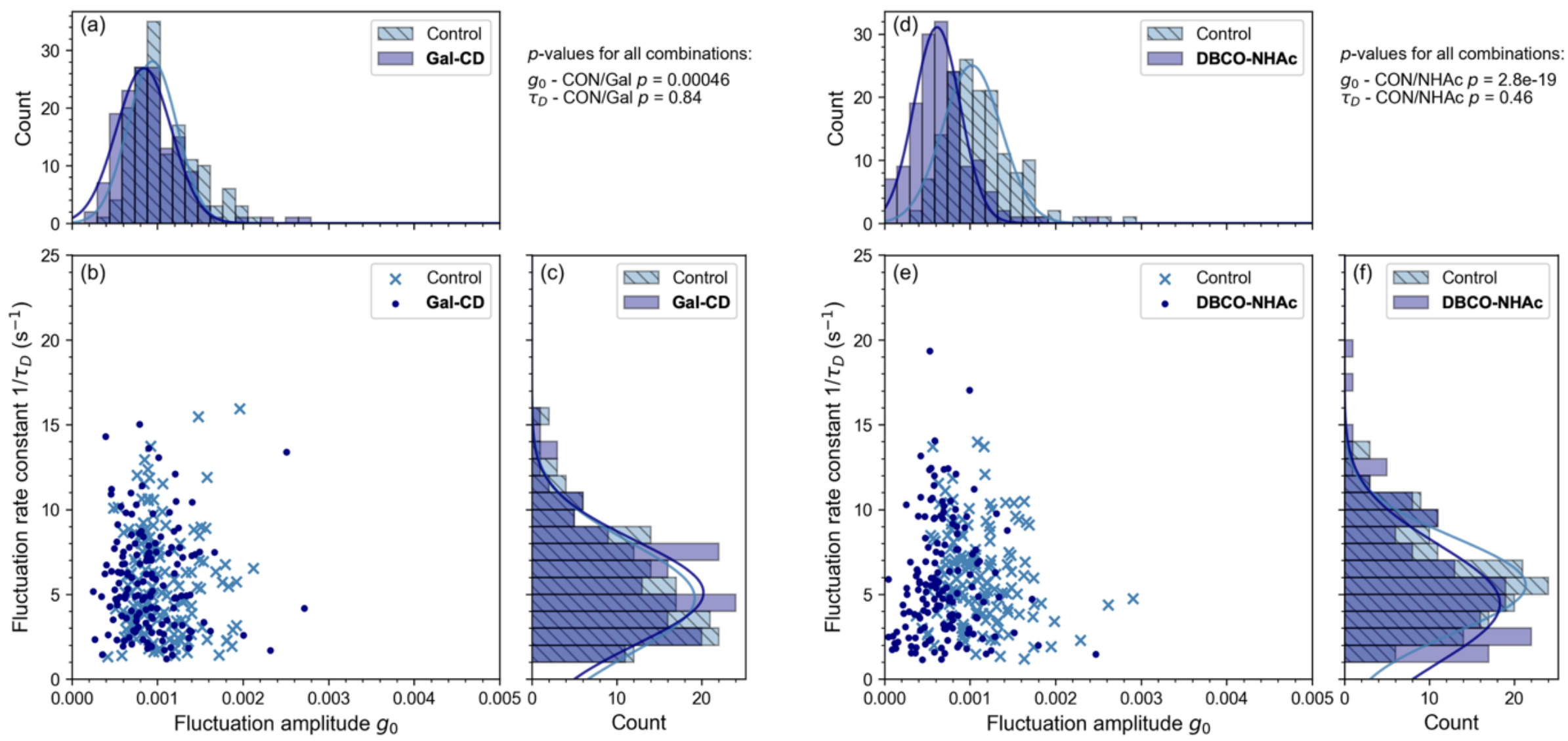

Figure S4: SCFI fluctuation data for *S. aureus* SH1000 exposed to **Gal-CD** and **DBCO-NHAc**, alongside untreated controls. All groups exposed to 90 min. UV LED irradiation including control samples. (a, d) Comparison between distributions of g_0 for control and **Gal-CD** / **DBCO-NHAc** treated suspensions. (b, e) Scatter plot of fluctuation amplitude (g_0) vs fluctuation rate ($1/\tau_D$) for control and **Gal-CD** / **DBCO-NHAc** treated suspensions. (c, f) Comparison between distributions of $1/\tau_D$ for control and **Gal-CD** / **DBCO-NHAc** treated suspensions. Each sample group contains 150 cells, three triplicate repeats (each a group of 50 cells) that have been pooled together.

References

(1) Garcia-Millan, T.; Ramos-Soriano, J.; Ghirardello, M.; Liu, X.; Santi, C. M.; Eloi, J. C.; Pridmore, N.; Harniman, R. L.; Morgan, D. J.; Hughes, S.; et al. Multicolor Photoluminescent Carbon Dots a La Carte for Biomedical Applications. *ACS Appl Mater Interfaces* **2023**. DOI: 10.1021/acsami.3c08200.
(2) Chio, T. I.; Gu, H.; Mukherjee, K.; Tumey, L. N.; Bane, S. L. Site-Specific Bioconjugation and Multi-Bioorthogonal Labeling via Rapid Formation of a Boron-Nitrogen Heterocycle. *Bioconjug Chem* **2019**, *30* (5), 1554-1564. DOI: 10.1021/acs.bioconjchem.9b00246.
(3) Parry, B. R.; Surovtsev, I. V.; Cabeen, M. T.; O'Hern, C. S.; Dufresne, E. R.; Jacobs-Wagner, C. The bacterial cytoplasm has glass-like properties and is fluidized by metabolic activity. *Cell* **2014**, *156* (1-2), 183-194. DOI: 10.1016/j.cell.2013.11.028.
(4) Rehnstrom, H.; Eley, A.; Clayton, N. S.; Plant, G.; Oviedo-Chavez, D.; Ede, B.; Hill, D. J.; Ridley, A. J.; Antognozzi, M. A rapid method of evaluating cytotoxic drug efficacy using sub-cellular fluctuation imaging. *Sci Rep* **2025**, *16* (1), 723. DOI: 10.1038/s41598-025-30295-9.
(5) a) Krieger, J. W.; Singh, A. P.; Bag, N.; Garbe, C. S.; Saunders, T. E.; Langowski, J.; Wohland, T. Imaging fluorescence (cross-) correlation spectroscopy in live cells and organisms. *Nat Protoc* **2015**, *10* (12), 1948-1974. DOI: 10.1038/nprot.2015.100 b) Kannan, B.; Guo, L.; Sudhaharan, T.; Ahmed, S.; Maruyama, I.; Wohland, T. Spatially resolved total internal reflection fluorescence correlation microscopy using an electron multiplying charge-coupled device camera. *Anal Chem* **2007**, *79* (12), 4463-4470. DOI: 10.1021/ac0624546.
(6) Kannan, B.; Har, J. Y.; Liu, P.; Maruyama, I.; Ding, J. L.; Wohland, T. Electron multiplying charge-coupled device camera based fluorescence correlation spectroscopy. *Anal Chem* **2006**, *78* (10), 3444-3451. DOI: 10.1021/ac0600959 .
(7) Szeremeta, W. K.; Harniman, R. L.; Bermingham, C. R.; Antognozzi, M. Towards a Fully Automated Scanning Probe Microscope for Biomedical Applications. *Sensors* **2021**, *21* (9), 3027. DOI: 10.3390/s21093027.
